# Non-coronary arterial outcomes in people with type 1 diabetes mellitus: a Swedish retrospective cohort study

**DOI:** 10.1016/j.lanepe.2024.100852

**Published:** 2024-02-15

**Authors:** Tarik Avdic, Björn Eliasson, Araz Rawshani, Jan Boren, Hertzel C. Gerstein, Joakim Nordanstig, Mohamad Rihawi, Joshua A. Beckman, Darren K. McGuire, Elmir Omerovic, Naveed Sattar, Deepak L. Bhatt, Aidin Rawshani

**Affiliations:** a Sahlgrenska Academy, Gothenburg, Sweden; b Department of Medicine, Sahlgrenska University Hospital Gothenburg, Sweden; c Department of Molecular and Clinical Medicine, Institute of Medicine, University of Gothenburg, Sweden; d Wallenberg Laboratory for Cardiovascular and Metabolic Research, Institute of Medicine, University of Gothenburg, Sweden; e Population Health Research Institute, McMaster University and Hamilton Health Sciences, Hamilton, ON, Canada; f Department of Vascular Surgery at the Sahlgrenska University Hospital, Gothenburg, Sweden; g Division of Vascular Medicine, Department of Internal Medicine, University of Texas Southwestern Medical Center, Dallas, TX, USA; h Division of Cardiology, Department of Internal Medicine, University of Texas Southwestern Medical Center, Dallas, TX, USA; i Institute of Cardiovascular and Medical Sciences, British Heart Foundation Glasgow Cardiovascular Research Centre Division of Cardiology, United Kingdom; j Mount Sinai Fuster Heart Hospital, Icahn School of Medicine at Mount Sinai, New York, NY, USA; k Parkland Health, Dallas, TX, USA; l The Lundberg Laboratory for Diabetes Research, Department of Molecular and Clinical Medicine, University of Gothenburg

**Keywords:** Type 1 diabetes mellitus, Non-coronary complications, Lower extremity artery disease, Extracranial large artery disease, Aortic aneurysm, Diabetic foot syndrome

## Abstract

**Background:**

Observational studies on long-term trends, risk factor association and importance are scarce for type 1 diabetes mellitus and peripheral arterial outcomes. We set out to investigate trends in non-coronary complications and their relationships with cardiovascular risk factors in persons with type 1 diabetes mellitus compared to matched controls.

**Methods:**

34,263 persons with type 1 diabetes mellitus from the Swedish National Diabetes Register and 164,063 matched controls were included. Incidence rates of extracranial large artery disease, aortic aneurysm, aortic dissection, lower extremity artery disease, and diabetic foot syndrome were analyzed using standardized incidence rates and Cox regression.

**Findings:**

Between 2001 and 2019, type 1 diabetes mellitus incidence rates per 100,000 person-years were as follows: extracranial large artery disease 296.5–84.3, aortic aneurysm 0–9.2, aortic dissection remained at 0, lower extremity artery disease 456.6–311.1, and diabetic foot disease 814.7–77.6. Persons with type 1 diabetes mellitus with cardiometabolic risk factors at target range did not exhibit excess risk of extracranial large artery disease [HR 0.83 (95% CI, 0.20–3.36)] or lower extremity artery disease [HR 0.94 (95% CI, 0.30–2.93)], compared to controls. Persons with type 1 diabetes with all risk factors at baseline, had substantially elevated risk for diabetic foot disease [HR 29.44 (95% CI, 3.83–226.04)], compared to persons with type 1 diabetes with no risk factors. Persons with type 1 diabetes mellitus continued to display a lower risk for aortic aneurysm, even with three cardiovascular risk factors at baseline [HR 0.31 (95% CI, 0.15–0.67)]. Relative importance analyses demonstrated that education, glycated hemoglobin (HbA1c), duration of diabetes and lipids explained 54% of extracranial large artery disease, while HbA1c, smoking and systolic blood pressure explained 50% of lower extremity artery disease and HbA1c alone contributed to 41% of diabetic foot disease. Income, duration of diabetes and body mass index explained 66% of the contribution to aortic aneurysm.

**Interpretation:**

Peripheral arterial complications decreased in persons with type 1 diabetes mellitus, except for aortic aneurysm which remained low. Besides glycemic control, traditional cardiovascular risk factors were associated with incident outcomes. Risk of these outcomes increased with additional risk factors present. Persons with type 1 diabetes mellitus exhibited a lower risk of aortic aneurysm compared to controls, despite presence of cardiovascular risk factors.

**Funding:**

Swedish Governmental and the county support of research and education of doctors, the 10.13039/501100003793Swedish Heart and Lung Foundation, Sweden and Åke-Wibergs grant.

**Research in context**

**Evidence before this study**

Type 1 diabetes is associated with increased rates and higher risk of non-coronary complications. To investigate this further, we conducted a comprehensive search on PubMed from the inception of the database until April 1, 2023. Our search employed the terms “peripheral arterial disease AND type 1 diabetes AND incidence OR risk”. For each search, the term “peripheral arterial disease” was substituted with one of the specific outcomes evaluated in our study. There is limited evidence regarding the rates and risk associations between type 1 diabetes, cardiometabolic risk factors, and atherosclerotic and non-atherosclerotic non-coronary complications within a well-defined cohort with a follow-up period spanning two decades. Although numerous studies have examined rates, excess risk, and association of cardiometabolic risk factors related to peripheral arterial disease, these studies predominantly focused on large-vessel peripheral arterial disease and were not able to differentiate between diabetes types. The examination of rates, excess risk and modifiable risk factors for the entire peripheral arterial tree in persons with type 1 diabetes remains largely unexplored.

**Added value of this study**

In a large observational study conducted between 2000 and 2019, we compared individuals with type 1 diabetes to matched controls from the general population. Our findings reveal a decline in rates of atherosclerotic non-coronary complications, whereas cases of aortic aneurysm have not changed in persons with type 1 diabetes. Notably, small-vessel peripheral arterial disease has rejoiced the greatest rates and risk reduction out of all non-coronary complications. Regarding risk factors, maintaining glycated hemoglobin and systolic blood pressure levels below the recommended therapeutic targets significantly reduces the risk of atherosclerotic non-coronary complications. Among various lipids, low-density lipoprotein cholesterol levels demonstrated the greatest risk reduction. Multifactorial risk factor analyses demonstrated a stepwise increase in risk for atherosclerotic non-coronary complications with each risk factor not within the therapeutic target range. However, optimal risk factor control at baseline resulted in a minimal increase in risk compared to matched controls. Interestingly, poor risk factor control was associated with a reduced risk for aortic complications. To achieve the most substantial relative risk reduction for all non-coronary complications, it is crucial to focus on improving glycated hemoglobin levels, blood pressure control, and smoking cessation. These interventions hold the greatest potential for reducing the risk associated with non-coronary complications.

**Implications of all the available evidence**

Cardiovascular disease is undergoing a gradual shift, where the focus is shifting from central cardiovascular complications to the peripheral arterial tree. Among non-coronary complications, small-vessel peripheral arterial disease has emerged as the predominant condition. Our study highlights the potential for even greater risk reduction in future events through the maintenance of lower levels of cardiometabolic risk factors. Furthermore, we observed that the relative importance of these risk factors differs between cardiovascular disease and non-coronary complications, with glycated hemoglobin levels assuming a more significant role in the latter.

## Introduction

Type 1 diabetes mellitus presents a significant risk for developing cardiovascular disease (CVD),^1^, ^2^, ^3^ and is associated with a 3–10 fold higher risk for myocardial infarction or cardiovascular death compared to the general population.^3^^,^^4^ Previous analyses based on two decades of data from the Swedish National Diabetes Register (NDR) have revealed a decline in the incidence rates of CVD, such as ischemic heart disease, heart failure, cerebrovascular disease and diabetic nephropathy, among individuals with type 1 diabetes mellitus.^5^^,^^6^ These declines have been attributed to improved management of dyslipidemia, blood pressure, hyperglycemia, as well as improved organized care for individuals with type 1 diabetes mellitus.^7^, ^8^, ^9^, ^10^ Whether similar changes in rates are occurring for outcomes related to non-coronary complications has not been carefully studied.

The association between type 1 diabetes mellitus and a diverse array of non-coronary complications and complications, including extracranial large artery disease (extracranial large artery disease), aortic aneurysm, aortic dissection, lower extremity artery disease and diabetic foot syndrome, i.e., distal microangiopathy and neuropathy, remains unclear. Previous studies have primarily focused on limited aspects of these disease outcomes.^11^^,^^12^ Rates of diabetic foot disease are suggested to higher than severe amputations, but data are inconsistent and limited.^13^^,^^14^ Multifactorial intervention is recommended to prevent progression of diabetic foot disease.^15^ The objective of this study was to 1) to analyze long-term trends in the incidence rates of first-events for extracranial large artery disease, aortic aneurysm, aortic dissection, lower extremity artery disease, and diabetic foot disease over the past two decades; 2) to model the relative predictive importance of various risk factors for each of these outcomes among individuals with type 1 diabetes mellitus; 3) to examine the association of maintaining cardiometabolic risk factors within target range and risk of developing outcomes, compared with matched controls and 4) to determine the “optimal” levels of selected cardiometabolic risk factors based on statistical associations among individuals with type 1 diabetes mellitus concerning the aforementioned disease outcomes.

## Methods

### Study design

This registry study was approved by the Swedish Ethical Review Authority (reference number: 2020–04796).

### Data sources and study cohort

The primary source of data for this study was the NDR, which has been extensively described in previous literature.^5^^,^^16^ The diagnosis of type 1 diabetes mellitus primarily relied on an epidemiological definition of type 1 diabetes mellitus, which classifies individuals as having developed diabetes before the age of 30 and requiring insulin treatment exclusively. Furthermore, a small subgroup of diabetes participants (fewer than 4500) had missing data in terms of their diabetes classification, as per the epidemiological definition. If these individuals had their diabetes status determined through clinical assessments conducted by physicians, and were identified as type 1 diabetes mellitus patients, they were also included in the diabetes cohort.^17^ The study comprised of individuals with type 1 diabetes mellitus who had at least one entry recorded in the NDR between January 1st, 2001, and December 31st, 2019. Each participant with type 1 diabetes mellitus was, at initial registration in the NDR, matched to five age-, sex- and county-matched control persons from the general population. This data was retrieved from the governmental agency called Statistics Sweden (SCB).

### Outcomes

The study encompassed five outcomes: extracranial large artery disease (atherosclerosis in carotid and vertebral arteries, i.e. both ischemic stroke and stenosis by imaging study), aortic aneurysm (thoracic, thoracoabdominal, and abdominal locations), aortic dissection (Stanford Type A and Type B aortic dissection), lower extremity artery disease (atherosclerosis from the infrarenal aorta to leg arteries), and diabetic foot disease (includes microangiopathy and neuropathy, ICD-codes for diabetic foot syndrome-related microvascular complications).

Identification of these outcomes was based on the main diagnosis code and up to six secondary codes derived from the Swedish National Patient Register (In- and Outpatient Register), utilizing the International Classification of Diseases (ICD) 10-codes.^18^ All outcomes were equally assessed and confirmed in both persons with type 1 diabetes mellitus and in controls. The specific codes utilized for defining each disease outcome can be found in Supplementary Table S1.

### Construction of final-cohort and follow-up procedures

After merger of the data for matched controls with our dataset of unique baseline data on type 1 diabetes mellitus participants, we applied our exclusion criteria to construct a final cohort without any peripheral arterial complication at baseline. Study participant with any of the non-coronary complications at baseline, i.e., prior to their inclusion in the registry (NDR), were excluded from the study. We performed a separate exclusion approach for cases with diabetes and controls. Participants with type 1 diabetes mellitus who met any exclusion criterion were excluded from the analyses along with their corresponding matched controls. In those cases, where a matched control had developed an event prior to their inclusion in the registry, and their counterpart with type 1 diabetes mellitus had not, the control was excluded individually, without exclusion of their matched counterpart with type 1 diabetes mellitus. This method has been used in previous publications.^5^^,^^6^^,^^8^^,^^11^ This results in a slightly lower matching ratio between cases with diabetes and controls (1:4,8 matching ratio) in the final cohort and slight variations in age and sex distribution between persons with diabetes and controls. For each analysis, study participants were followed until end of study time, or until an event or death occurred, whichever came first (Supplementary Fig. S1 Flowchart).

### Statistical analyses

Descriptive statistics are presented in terms of mean values with standard deviation for continuous variables and counts with percentages for categorical variables. The multivariable regression models included several variables, such as: age, sex, baseline comorbidities (hypertension, chronic obstructive pulmonary disease, ischemic heart disease, heart failure, dementia, cerebrovascular disease, end-stage kidney disease, and cancer), medication use (statins, anticoagulant medications, antithrombotic medication and anti-hypertensive medication), socioeconomic variables (individual income level, educational level, ethnicity (i.e., Scandinavian vs non-Scandinavian) and civil status) and CV risk factors. Information on CV risk factors were only available for persons with type 1 diabetes mellitus, and consisted of: glycated hemoglobin (HbA1c), systolic (SBP) and diastolic blood pressure (DBP), low-density lipoprotein cholesterol (LDL-C), high-density lipoprotein cholesterol (HDL-C), total cholesterol, triglycerides, diabetes duration, body mass index (BMI), estimated glomerular filtration rate (eGFR) and smoking data. Please see more detailed description of the multivariable regression modeling in the statistical methods section of the data supplement.

**Fig. 1:**
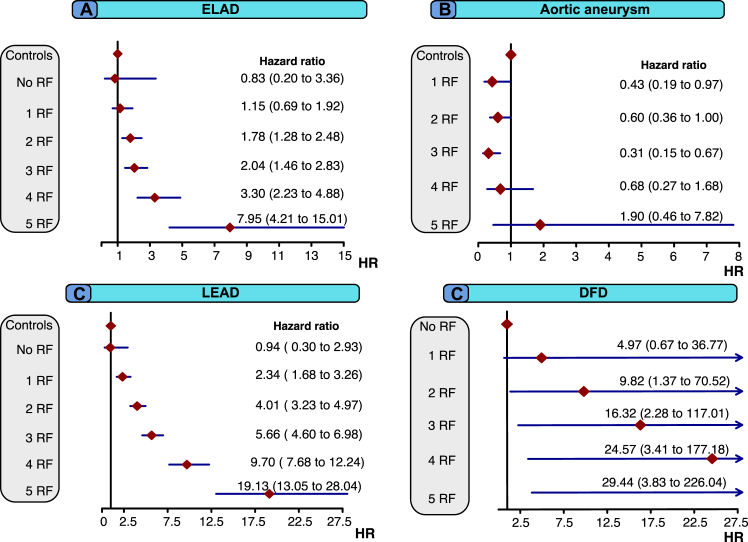
Adjusted hazard ratios for all outcomes, according to number of cardiovascular risk factors beyond target in people with type 1 diabetes mellitus, compared with matched controls. Legend: People with type 1 diabetes mellitus are stratified into six different subgroups based on the number of risk factors, ranging from 0 to 5, beyond target.

**Fig. 2:**
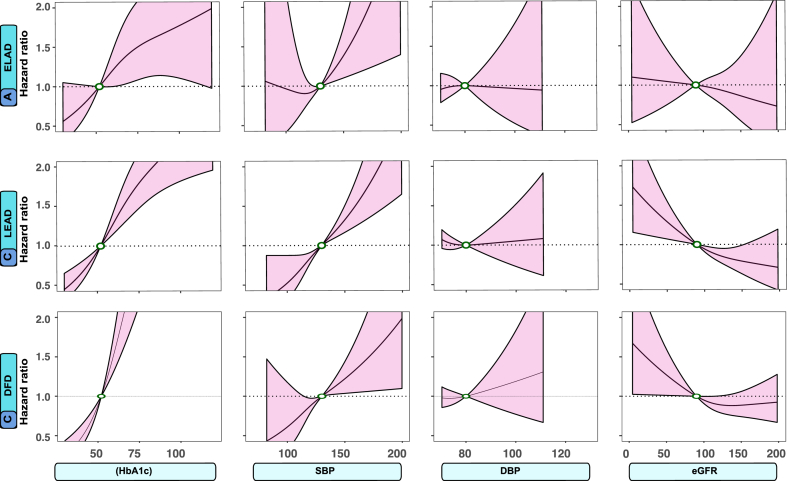
Association between risk factor levels and outcomes in persons with type 1 diabetes mellitus. Legend: To capture potential non-linear associations, the Cox regression models incorporated restricted cubic splines with three evenly spaced knots The dark lines indicate the hazard function and the shaded areas 95% confidence intervals. Continuous variables were modeled with restricted cubic splines. The following cut-off levels were used for risk factors: glycated hemoglobin (≥7.0% (52 mmol/mol)), SBP (≥130 mmHg), DBP (≥80 mmHg), and eGFR (≤90 ml/min/1.73 m^2^).

**Fig. 3:**
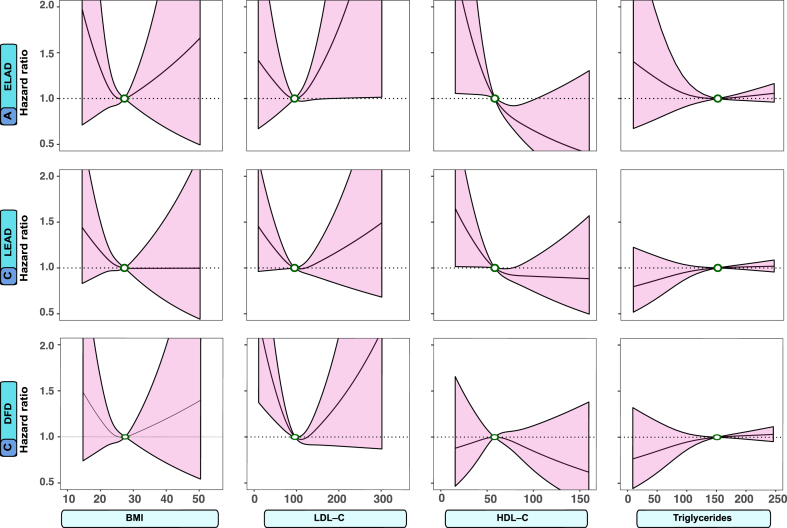
Association between risk factor levels and outcomes in persons with type 1 diabetes mellitus. Legend: The dark lines indicate the hazard function and the shaded areas 95% confidence intervals. The following cut-off levels were used for risk factors: BMI ≥27.5 kg/m^2^, LDL-C (≥96 mg/dL), HDL-C (≤60 mg/dL), and triglycerides (≥151 mg/dL).

**Fig. 4:**
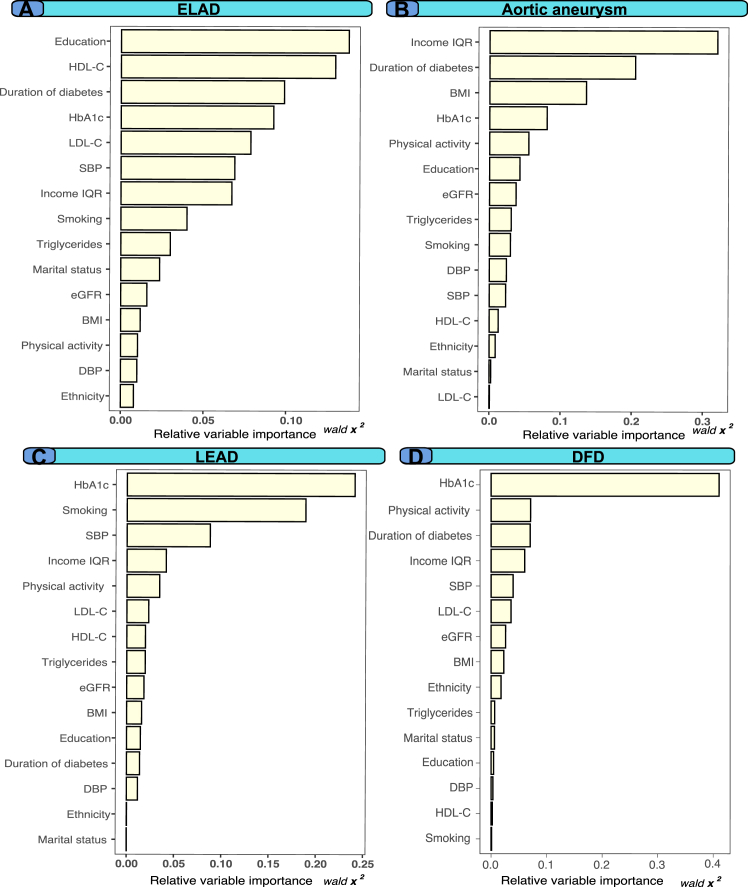
Relative importance of variables generated from modified Cox proportional hazards models for all outcomes in people with type 1 diabetes mellitus. Legend: Variables with a high importance estimate have a higher partial graded effect predictive to the model and are deemed important.

Matched control persons are used in the calculation of rates and regression models in Fig. 1. Information regarding cardiometabolic risk factors in individuals with type 1 diabetes mellitus is utilized in the regression models for the analyses presented in Fig. 1, Fig. 2, Fig. 3, Fig. 4. In Fig. 2, Fig. 3, Fig. 4, these variables are represented as continuous variables, whereas Fig. 1 employs categorized predictors for cardiometabolic risk factors.

### Standardized incidence rates, change in risk over time and all-cause mortality after incident peripheral artery outcome

**Fig. 5:**
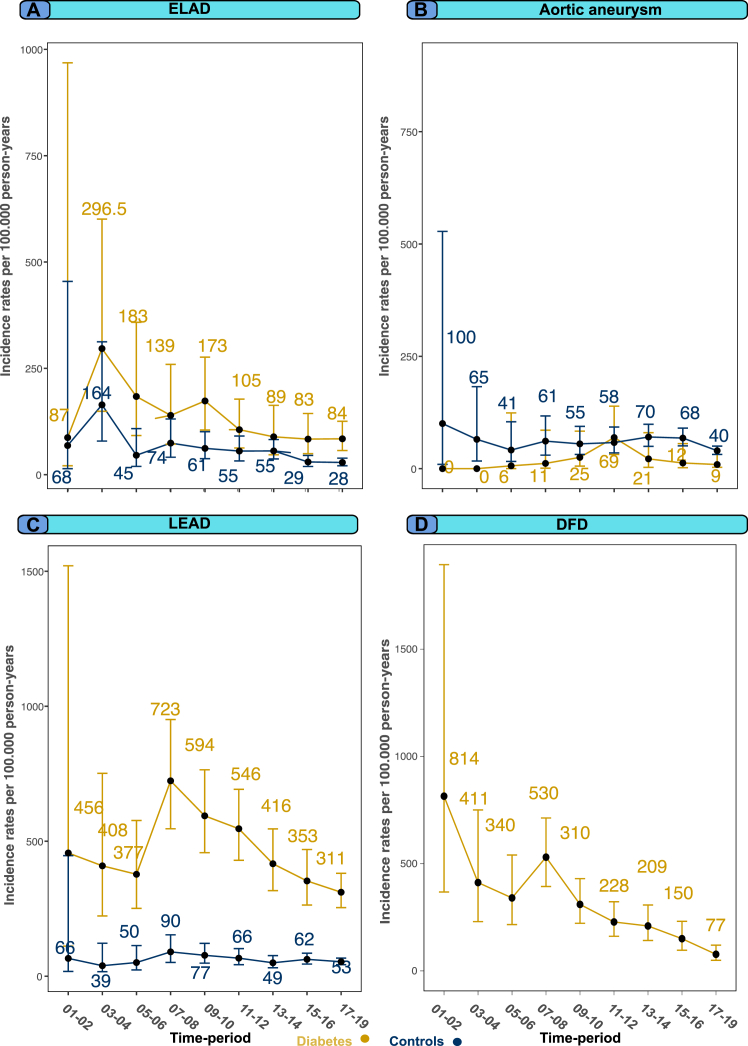
Standardized incidence rates for all outcomes among people with type 1 diabetes mellitus and matched controls. Legend: Age- and sex standardized incidence rates for all outcomes in people with type 1 diabetes mellitus compared with matched controls from the general population. Abbreviations: extracranial large artery disease = Extracranial large artery disease, LEAD = Lower extremity artery disease, diabetic foot disease = Diabetic foot syndrome.

The study spanned from 2001 to 2019, and the duration was divided into 2-year periods, with the exception of the final 3-year period (Fig. 5). To calculate incidence rates, the number of events that occurred during each study period (numerator) was divided by the number of study participants at risk during the same period (denominator) and reported as rates per 100,000 person-years. The incidence rates were standardized directly to the age and sex distribution of the initial study period. Participants that die during the follow-up, express a desire to withdraw from the NDR, or undergo changes in citizenship, are subject to exclusion. This exclusion is necessary as such individuals are unable to contribute person-years or events to the subsequent time periods. It is noteworthy that the incidence of participants leaving the registry is exceedingly rare, if not negligible. Cox regression models were also constructed to account for age, sex, and time period, aiming to assess the change of risk over time. Time period was incorporated as a categorical or continuous variable in these models. In the models where time period was treated as a continuous predictor, coefficients were exponentiated and raised to the power of 8. This approach employs an arbitrary number to estimate the cumulative linear change in risk over a span of 17.8 years and assumes a linear relationship between time and risk. Thus, there are two separate assessments of change in risk over the study period to provide nuances of the development of non-coronary complications. For all-cause mortality after incident peripheral artery outcomes, Kaplan-Meir survival curve for each outcome includes a subset of the cohort with study participants who had experienced a peripheral artery outcome during follow-up, and subtracted the survival time of death (death or end of follow-up) with the survival time calculated from each participants entry into the registry to development of a peripheral arterial outcome.

### Associations between risk factors and all studied outcomes

The Cox models were employed to examine the risk patterns between cardiometabolic risk factors and the outcomes of interest in individuals with type 1 diabetes mellitus. To capture potential non-linear associations, the Cox models incorporated restricted cubic splines with three evenly spaced knots. The models intend to display risk association in comparison to therapeutic target levels. The models took into account various risk factors, including glycated hemoglobin (HbA1c), systolic blood pressure (SBP), diastolic blood pressure (DBP), estimated glomerular filtration rate (eGFR), body mass index (BMI), low-density lipoprotein cholesterol (LDL-C), high-density lipoprotein cholesterol (HDL-C), and triglycerides (TG).

These models were also adjusted for age, sex, smoking status, physical activity level, ethnicity, socio-economic factors (marital status, income level, educational level), comorbidities, and pharmacological treatment.

### Cardiometabolic risk factor targets

Cox models were employed to investigate the risk of outcomes for individuals with type 1 diabetes mellitus that had one or several cardiometabolic risk factor within therapeutic target range, compared with matched controls. To achieve this, individuals with type 1 diabetes mellitus were stratified into six distinct subgroups based on the number of risk factors present beyond the recommended guideline target levels at baseline. The risk factors were treated as binary variables (yes/no) and included HbA1c (≥7.0%), blood pressure (SBP ≥130 mmHg and DBP ≥80 mmHg), current smoking, LDL-C (≥97 mg/dL), and the presence of micro- or macroalbuminuria.

These Cox models were adjusted for various factors including age, sex, socioeconomics (such as marital status, income level, and educational level), comorbidities, and pharmacological treatment. Control subjects were assigned a diabetes duration of 0 years, while individuals with type 1 diabetes mellitus had their diabetes duration centered around the grand mean. In the diabetic foot disease model, the reference group consisted of individuals with type 1 diabetes mellitus who had none of the analyzed risk factors beyond target levels, and diabetes duration was fully adjusted for.

### Relative importance of variables to the model for outcome risk

To determine the relative importance or the partial graded effect of each predictor to the regression model for each outcome, an extended application for Cox proportional hazards model with restricted cubic splines was employed. In this study, the relative importance was assessed by calculating the explainable log-likelihood attributable to each risk factor, and is presented as the Wald χ^2^ statistic minus the degrees of freedom for each covariate. The regression models also accounted for sex, age, comorbidities and pharmacological treatment. The time scale used in these models was attained age.

### Missing data

To address missing data, we used multiple imputation by chained equations (MICE). This approach allowed for the imputation of missing values based on observed data. The MICE method imputes missing values by prediction based on observed data. Each variable with missing data is imputed one at a time using iterative regression models. The imputed dataset's outcomes are combined into a final dataset, which serves as the basis for our analyses. The variables included in the imputation model can be found in Supplementary Table S2, providing transparency regarding the variables used in the imputation process.

In terms of statistical significance, a 2-sided p-value threshold of less than 0.05 was considered statistically significant, and all confidence intervals (CIs) were calculated at the 95% level. It is important to note that due to the explorative nature of the study, analyses results were not corrected for multiple comparisons.

Proportional hazards assumption holds for all Cox regression models. All statistical calculations were performed using R version R version 4.3.1 from the R Foundation for Statistical Computing, and the analyses were conducted in RStudio (Gothenburg, Sweden).

### Role of the funding source

This study is supported by grants from the Swedish state under an agreement between the Swedish government and the county councils concerning economic support of research and education of doctors [ALFGBG-966187], the Swedish Heart and Lung Foundation, Sweden [HLF 2019-0532] and Åke-Wibergs stiftelse [M22-0206].

## Results

### Baseline characteristics of study participants

This study included a cohort of 34,263 individuals with type 1 diabetes mellitus and 164,063 matched controls. The mean age of participants in the group with type 1 diabetes mellitus was 33.4 years of age at baseline, slightly higher than the control group with a mean age of 32.1 years. In terms of sex distribution, 43.3% of the cohort with type 1 diabetes mellitus were women. The median follow-up time for individuals with type 1 diabetes mellitus was 10.16 years. Baseline characteristics for patients with type 1 diabetes mellitus and controls are presented in Table 1 and Supplementary Table S3, while baseline characteristics for type 1 diabetes mellitus according to time period are presented in Supplementary Table S4.

**Table 1:** Baseline characteristics for patients with type 1 diabetes and matched controls according to age-categories.

	Diabetes: <45	Controls: <45	Diabetes: 45–54	Controls: 45–54	Diabetes: 55–64	Controls: 55–64	Diabetes: 65–74	Controls: 65–74	Diabetes: >75	Controls: >75
n	26,215	130,838	2649	12,025	3999	17,284	899	2677	501	1239
Sex = female %)	11,081 (42.3)	55,605 (42.5)	1171 (44.2)	5547 (46.1)	1855 (46.4)	8430 (48.8)	434 (48.3)	1525 (57.0)	285 (56.9)	815 (65.8)
Age (mean (SD))	25.52 (7.65)	25.53 (7.61)	49.27 (2.86)	49.16 (2.85)	60.19 (2.38)	60.28 (2.27)	68.76 (2.75)	68.66 (2.74)	81.09 (4.96)	80.82 (4.85)
Education n (%)										
Post-secondary education ≥12 years	4596 (17.5)	26,108 (20.0)	809 (30.5)	3994 (33.2)	562 (14.1)	2226 (12.9)	192 (21.4)	617 (23.0)	68 (13.6)	181 (14.6)
Pre-secondary education ≤9 years	11,038 (42.1)	52,493 (40.1)	549 (20.7)	2186 (18.2)	2567 (64.2)	11,807 (68.3)	372 (41.4)	1037 (38.7)	292 (58.3)	684 (55.2)
Secondary education >9–12 years	10,581 (40.4)	52,237 (39.9)	1291 (48.7)	5845 (48.6)	870 (21.8)	3251 (18.8)	335 (37.3)	1023 (38.2)	141 (28.1)	374 (30.2)
Civil status: married n (%)	3268 (12.5)	16,982 (13.0)	1383 (52.2)	6594 (54.8)	1188 (29.7)	4434 (25.7)	591 (65.7)	1563 (58.4)	242 (48.3)	531 (42.9)
Ethnicity = Scandinavian, n (%)	24,237 (92.5)	118,116 (90.3)	2447 (92.4)	11,139 (92.6)	3262 (81.6)	14,738 (85.3)	849 (94.4)	2510 (93.8)	452 (90.2)	1155 (93.2)
Income family interquartile range (IQR) n (%)										
Quartile 1	6522 (24.9)	29,040 (22.2)	635 (24.0)	2081 (17.3)	488 (12.2)	1472 (8.5)	290 (32.3)	843 (31.5)	264 (52.7)	647 (52.2)
Quartile 2	5253 (20.0)	27,623 (21.1)	675 (25.5)	2754 (22.9)	588 (14.7)	1907 (11.0)	325 (36.2)	940 (35.1)	167 (33.3)	392 (31.6)
Quartile 3	5468 (20.9)	27,911 (21.3)	729 (27.5)	3400 (28.3)	2486 (62.2)	11,838 (68.5)	150 (16.7)	475 (17.7)	50 (10.0)	115 (9.3)
Quartile 4	8972 (34.2)	46,264 (35.4)	610 (23.0)	3790 (31.5)	437 (10.9)	2067 (12.0)	134 (14.9)	419 (15.7)	20 (4.0)	85 (6.9)
Income (interquartile range (IQR)) n (%)										
Quartile 1	16,503 (63.0)	78,492 (60.0)	563 (21.3)	1910 (15.9)	533 (13.3)	1458 (8.4)	379 (42.2)	993 (37.1)	255 (50.9)	596 (48.1)
Quartile 2	4508 (17.2)	21,889 (16.7)	863 (32.6)	3335 (27.7)	602 (15.1)	2028 (11.7)	281 (31.3)	834 (31.2)	181 (36.1)	446 (36.0)
Quartile 3	3163 (12.1)	17,694 (13.5)	694 (26.2)	3536 (29.4)	2465 (61.6)	11,938 (69.1)	118 (13.1)	438 (16.4)	40 (8.0)	109 (8.8)
Quartile 4	2041 (7.8)	12,763 (9.8)	529 (20.0)	3244 (27.0)	399 (10.0)	1860 (10.8)	121 (13.5)	412 (15.4)	25 (5.0)	88 (7.1)
Hypertension n (%)	688 (2.6)	267 (0.2)	377 (14.2)	223 (1.9)	489 (12.2)	461 (2.7)	290 (32.3)	332 (12.4)	217 (43.3)	309 (24.9)
Chronic obstructive pulmonary disease n (%)	8 (0.0)	36 (0.0)	10 (0.4)	34 (0.3)	30 (0.8)	76 (0.4)	18 (2.0)	63 (2.4)	32 (6.4)	48 (3.9)
Ischemic heart disease n (%)	52 (0.2)	47 (0.0)	128 (4.8)	109 (0.9)	255 (6.4)	271 (1.6)	166 (18.5)	187 (7.0)	153 (30.5)	188 (15.2)
Heart failure n (%)	28 (0.1)	44 (0.0)	34 (1.3)	33 (0.3)	80 (2.0)	66 (0.4)	61 (6.8)	56 (2.1)	118 (23.6)	88 (7.1)
Cerebrovascular disease n (%)	35 (0.1)	77 (0.1)	31 (1.2)	32 (0.3)	50 (1.3)	86 (0.5)	45 (5.0)	79 (3.0)	54 (10.8)	101 (8.2)
Dementia n (%)	3 (0.0)	3 (0.0)	1 (0.0)	3 (0.0)	9 (0.2)	8 (0.0)	6 (0.7)	17 (0.6)	20 (4.0)	64 (5.2)
End-stage renal disease n (%)	612 (2.3)	130 (0.1)	219 (8.3)	29 (0.2)	209 (5.2)	42 (0.2)	80 (8.9)	25 (0.9)	49 (9.8)	20 (1.6)
Cancer n (%)	170 (0.6)	587 (0.4)	73 (2.8)	284 (2.4)	122 (3.1)	499 (2.9)	104 (11.6)	324 (12.1)	107 (21.4)	227 (18.3)
Antihypertensive medication n (%)	5424 (20.7)	8043 (6.1)	1639 (61.9)	4113 (34.2)	1382 (34.6)	3495 (20.2)	349 (38.8)	1177 (44.0)	65 (13.0)	323 (26.1)
Statins n (%)	6126 (23.4)	2029 (1.6)	1692 (63.9)	1783 (14.8)	1354 (33.9)	1767 (10.2)	302 (33.6)	566 (21.1)	38 (7.6)	108 (8.7)
Antiocoagulant medication n (%)	383 (1.5)	1400 (1.1)	119 (4.5)	616 (5.1)	209 (5.2)	771 (4.5)	66 (7.3)	368 (13.7)	24 (4.8)	114 (9.2)
Antithrombotic medication n (%)	1279 (4.9)	1193 (0.9)	731 (27.6)	972 (8.1)	677 (16.9)	1138 (6.6)	192 (21.4)	446 (16.7)	35 (7.0)	130 (10.5)
Age at onset of diabetes (mean (SD))	16.51 (9.83)		23.92 (12.01)		22.06 (13.34)		34.40 (18.16)		48.83 (22.98)	
Duration of diabetes (mean (SD))	9.04 (8.15)		25.22 (12.32)		20.53 (16.48)		33.92 (17.92)		30.90 (20.63)	
Glycated hemoglobin levels (mean (SD)) (mmol/mol)	65.44 (19.07)		65.81 (15.34)		64.65 (18.10)		62.67 (14.93)		63.19 (17.33)	
Current smoking n (%)	3989 (15.2)		483 (18.2)		581 (14.5)		85 (9.5)		25 (5.0)	
Albuminuria n (%)										
No albuminuria	24,074 (91.8)		2064 (77.9)		3327 (83.2)		604 (67.2)		303 (60.5)	
Normal albuminuria	74 (0.3)		6 (0.2)		14 (0.4)		3 (0.3)		0 (0.0)	
Microalbuminuria	1517 (5.8)		356 (13.4)		410 (10.3)		190 (21.1)		112 (22.4)	
Macroalbuminuria	550 (2.1)		223 (8.4)		248 (6.2)		102 (11.3)		86 (17.2)	
Estimated glomerular filtration rate (eGFR) (mean (SD))	123.73 (56.45)		86.73 (23.89)		110.66 (68.62)		71.82 (25.47)		61.18 (24.49)	
Retinopathy n (%)	6200 (23.7)		1551 (58.6)		1815 (45.4)		585 (65.1)		284 (56.7)	
Systolic blood pressure (mean (SD)) (mmHg)	119.98 (12.88)		132.98 (16.93)		128.99 (18.07)		141.62 (17.64)		140.47 (19.46)	
Diastolic blood pressure (mean (SD)) (mmHg)	72.56 (9.02)		75.80 (9.14)		73.66 (9.21)		73.18 (9.56)		71.89 (10.65)	
Total cholesterol (mean (SD)) (mg/dL)	181.29 (41.59)		195.04 (42.11)		185.10 (42.96)		190.52 (42.82)		187.59 (44.19)	
High-density lipoprotein cholesterol (mean (SD)) (mg/dL)	60.31 (18.41)		68.78 (22.58)		65.46 (21.87)		71.40 (24.65)		71.37 (24.37)	
Triglycerides (mean (SD)) (mg/dL)	118.27 (109.60)		117.80 (104.25)		116.92 (124.80)		120.91 (138.26)		130.43 (138.93)	
Low-density lipoprotein cholesterol (mean (SD)) (mg/dL)	117.63 (38.14)		105.49 (36.60)		99.44 (35.29)		98.25 (37.02)		94.67 (39.66)	
Physical activity n (%)										
1 = Never	2570 (9.8)		377 (14.2)		563 (14.1)		177 (19.7)		148 (29.5)	
2 = <1 time/week	3513 (13.4)		422 (15.9)		575 (14.4)		137 (15.2)		83 (16.6)	
3 = 1–2 times/week	5651 (21.6)		563 (21.3)		773 (19.3)		192 (21.4)		81 (16.2)	
4 = 3–5 times/week	7484 (28.5)		611 (23.1)		1018 (25.5)		181 (20.1)		80 (16.0)	
5 = Daily	6997 (26.7)		676 (25.5)		1070 (26.8)		212 (23.6)		109 (21.8)	
S-creatinine (mean (SD)) (μmol/L)	67.39 (31.02)		84.03 (58.45)		77.06 (49.56)		92.07 (45.67)		103.83 (56.25)	
Body mass index (mean (SD)) (kg/m2)	24.89 (4.13)		25.75 (4.23)		25.38 (4.34)		25.58 (4.36)		25.57 (4.35)	

### Incidence rates and change in risk over time

For a detailed overview of the number of events during each time-period and the corresponding crude and standardized incidence rates presented as the number of events per 100,000 person-years may be found in Supplementary Table S5.

In terms of extracranial large artery disease, there was a decrease in the incidence rates over the study period for both individuals with type 1 diabetes mellitus and controls. Among individuals with type 1 diabetes mellitus, the incidence rate for extracranial large artery disease decreased from 296.5 to 84.3 cases per 100,000 person-years. Controls experienced a more significant proportional reduction, with rates decreasing from 68.5 to 28.7 cases. For aortic aneurysm, incidence rates exhibited a non-linear but stable pattern for both the population with type 1 diabetes mellitus and controls. There was a slight increase in the incidence rates of aortic aneurysm among individuals with type 1 diabetes mellitus (0–9.2 cases per 100,000 person-years), while controls showed a decrease in rates (100.4–40.0 per 100,000 person-years). For aortic dissection, there was no change in rates for the cohort with type 1 diabetes mellitus (0–0 cases per 100,000 person-years) but a reduction for controls (311.1–63.8 cases per 100,000 person-years). Incidence rates for lower extremity artery disease in persons with type 1 diabetes mellitus decreased from 456.5 to 311.1 cases per 100,000 person-years. On the other hand, controls had relatively stable rates, ranging from 66.1 to 53.7 cases per 100,000 person-years. The incidence rates of diabetic foot disease dropped from 814.7 to 77.6 cases per 100,000 person-years. Cox models that assess change in risk over time reveals that risk for extracranial large artery disease between first and last time periods were HR 0.16 (95% CI, 0.04–0.52), and the roughly 18 years of change in hazard ratio was 0.18 (95% CI, 0.08–0.39), Supplementary Fig. S2 Panel A). Substantial relative risk reduction was observed for lower extremity artery disease and DFS, as well (Supplementary Fig. S2 Panel C and Panel D).

### Associations between selected risk factors and outcomes

The associations between selected cardiometabolic risk factors and extracranial large artery disease, lower extremity artery disease, and diabetic foot disease, respectively, in individuals with type 1 diabetes mellitus are presented in Figs. 2 and 3. For aortic aneurysm and aortic dissection, there were insufficient number of events to perform these analyses. The results demonstrate that levels of HbA1c and SBP above the guideline-recommended targets are associated with a higher risk of extracranial large artery disease and lower extremity artery disease (Fig. 2). LDL-C and BMI demonstrated a U-shaped pattern for extracranial large artery disease and lower extremity artery disease (Fig. 3). Levels of HbA1c and SBP above the recommended target levels were associated with a higher risk of diabetic foot disease (Fig. 2).

### Cardiometabolic risk factor levels and risk of outcomes, and excess risk

The adjusted hazard ratios for outcomes according to level of cardiometabolic risk factor within target range are presented in Fig. 4, panels A–D. The findings revealed a graded association between the increasing number of risk factors beyond the recommended targets levels and risk of incident extracranial large artery disease and lower extremity artery disease among individuals with type 1 diabetes mellitus. Individuals with type 1 diabetes mellitus who had none of the five analyzed risk factors outside target showed no excess risk of extracranial large artery disease (HR 0.82; 95% CI, 0.2–3.32) or lower extremity artery disease (HR 0.98; 95% CI, 0.31–3.07) compared with matched controls. However, those with all five risk factors outside target had significantly higher adjusted HRs for extracranial large artery disease (7.95; 95% CI, 4.21–15.01) and lower extremity artery disease (19.13; 95% CI, 13.05–28.04). Regarding aortic aneurysm, the risk was lower in individuals with type 1 diabetes mellitus who had 0–3 risk factors beyond target compared with controls. Ancillary analyses of abdominal and thoracic aortic aneurysm showed that the risk of both conditions was lower in individuals with type 1 diabetes mellitus compared with controls, even in the presence of several risk factors outside target (Supplementary Fig. S3).

For diabetic foot disease, the reference variables were individuals with type 1 diabetes mellitus who had no risk factors beyond target levels at baseline, compared with those with 1 or more risk factors at baseline. We observed a graded association between the number of risk factors that were not within target levels, and the risk of incident diabetic foot disease was substantially higher for those with poor risk factor control. Those with all five risk factors beyond the target had the highest risk of diabetic foot disease (HR 29.44; 95 % CI, 3.83–226.04).

The excess risk of selected outcomes for individuals with type 1 diabetes mellitus compared with matched controls, were as follows: extracranial large artery disease: HR 1.88 (95 % CI, 1.53–2.31); aortic aneurysm; HR 0.29 (95 % CI, 0.20–0.41); aortic dissection; HR 0.36 (95% CI, 0.14–0.94); and lower extremity artery disease: HR 6.28 (95% CI, 5.42–7.27) (Supplementary Table S6).

### Relative importance of risk factors for modeling outcomes

Fig. 5, panels A–D provide a comprehensive assessment of the relative importance of each variable to the models associated with extracranial large artery disease, lower extremity artery disease, aortic aneurysm, and diabetic foot disease outcomes in individuals with type 1 diabetes mellitus. In particular, HbA1c and SBP emerged as the variables with the greatest relative importance for the models of both extracranial large artery disease and lower extremity artery disease. Furthermore, the analyses identified current smoking as a predictor highly influential to the model for lower extremity artery disease. Dyslipidemia also exhibited significant relative importance to the model for extracranial large artery disease. HbA1c and diabetes duration demonstrated the strongest relative importance in the modeling for diabetic foot disease, contributing to almost 50% of the explanation of the Cox model for diabetic foot disease.

### All-cause mortality after incident peripheral arterial complication

**Fig. 6:**
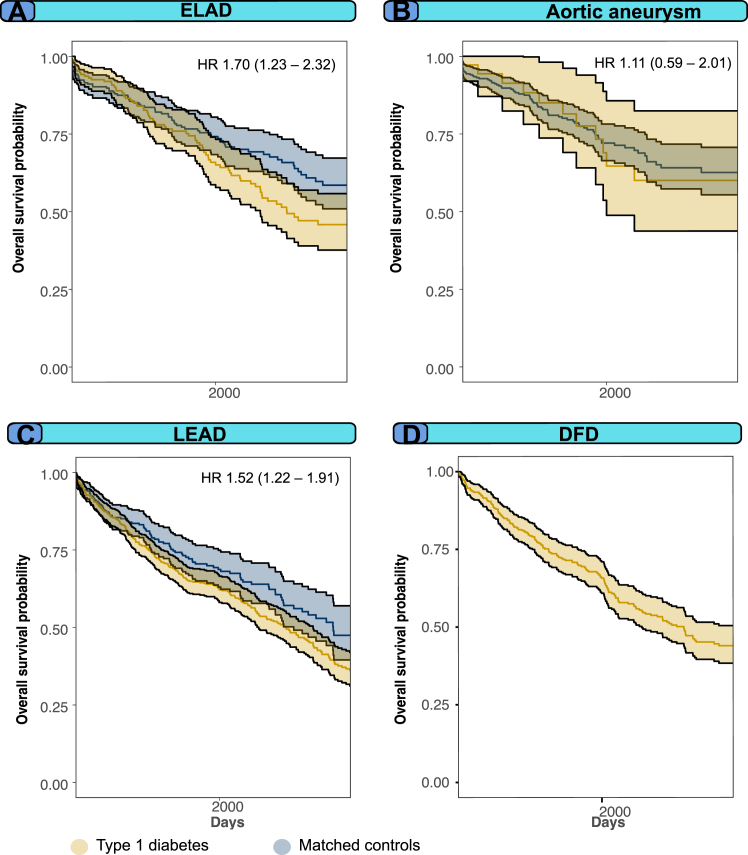
Survival curves after incident large- and small-vessel artery disease in both people with type 1 diabetes and matched controls. Legend: Survival curves after incident large- and small-vessel artery disease between index and 10 years.

Fig. 6, panels A–D display the survival curves for all-cause mortality following non-cardiovascular vascular arterial complications. The survival curves reveal a considerable mortality rate of approximately 40–60% within 10 years following the occurrence of any of the study outcomes, with the highest mortality rates in individuals with lower extremity artery disease.

### Changes in cardiometabolic risk factor levels

**Fig. 7:**
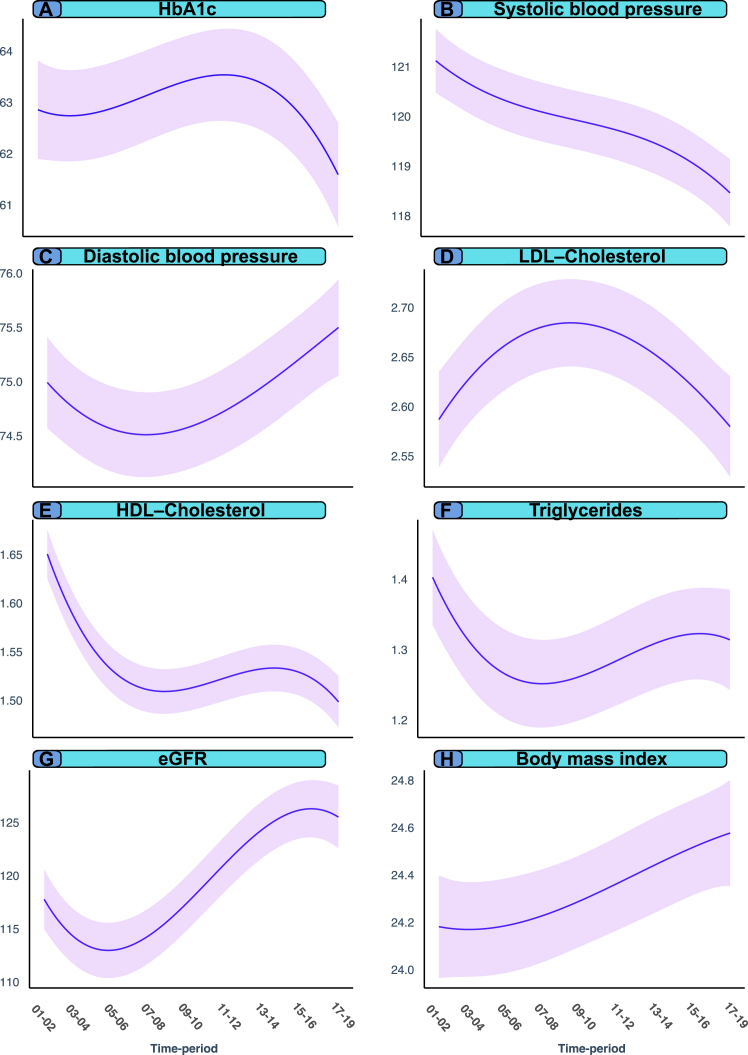
Changes in baseline levels of cardiometabolic risk factors in patients with type 1 diabetes, according to time of inclusion into the registry. Legend: Least-squared means of cardiometabolic risk factor levels adjusted for several covariates such as age, sex, socioeconomic variables, medications, comorbidities and risk factors.

Fig. 7 shows that HbA1c, SBP and eGFR are the only cardiometabolic risk factors in persons with type 1 diabetes mellitus that displayed an improvement over time, whereas DBP, all lipid parameters and BMI have worsened at onset of disease, over time.

## Discussion

In this observational study conducted from 2001 to 2019, significant reductions in the incidence rates of extracranial large artery disease, lower extremity artery disease and diabetic foot disease, were observed among individuals with type 1 diabetes mellitus and the general population, while aortic aneurysm and aortic dissection had an extremely low incidence in the cohort with type 1 diabetes mellitus. The most notable reductions were seen in extracranial large artery disease, lower extremity artery disease and diabetic foot disease. For diabetic foot disease there was a 10-fold decrease in absolute rates during two decades of follow-up. Despite these reductions, the incidence rates of lower extremity artery disease remained consistently high throughout the entire duration of the study, making it the most prevalent among all non-coronary complications. Rates for aortic aneurysm remained low and unchanged in type 1 diabetes mellitus but decreased among controls. Incident cases of aortic dissection were virtually non-existent in individuals with type 1 diabetes mellitus and in controls.

The observed reductions in rates of extracranial large artery disease, lower extremity artery disease and diabetic foot disease in individuals with type 1 diabetes mellitus are likely attributed to advancements in organized and developed care for this patient population.^2^^,^^7^^,^^9^^,^^19^ This includes more targeted cardiometabolic risk factor surveillance, stringent risk factor control, improved lifestyle interventions, and pharmacological treatments for risk factor control.^7^^,^^20^, ^21^, ^22^ The slight increase in incidence rates of aortic aneurysm among individuals with type 1 diabetes mellitus may be associated with the introduction of the abdominal aortic aneurysm screening program in Sweden in 2005.^23^ However, it is important to note that during the same period, the overall incidence of abdominal aortic aneurysm in Sweden has decreased.^24^ Our results also suggest that the significant reduction in rates of diabetic foot disease is strongly correlated with improved glycemic control. The results underscore a pivotal role of dysglycemia as the key risk factor for the development of diabetic distal microangiopathy and neuropathy.

The results from the relative importance analyses, and risk factor pattern analyses, for extracranial large artery disease and lower extremity artery disease support the critical role of hyperglycemia but also point out smoking, dysglycemia, and elevated systolic blood pressure as important risk factors, whereas dysglycemia is virtually the only explaining predictor for diabetic foot disease.^25^, ^26^, ^27^, ^28^ When comparing the importance of risk factors for incident lower extremity artery disease and extracranial large artery disease in individuals with type 1 diabetes mellitus with previous results on risk factor importance for traditional CVD outcomes,^29^ it is evident that the risk factor profile may vary.

Beyond the conventional cardiometabolic risk factors, it is imperative to incorporate duration of diabetes into the assessment of cardiovascular risk stratification among individuals with type 1 diabetes mellitus, as emphasized in the referenced Australian study. In the present investigation, the duration of diabetes is included as a continuous variable within regression models, that includes cardiometabolic risk factors.^30^ Thus, the results of the present study provide important insights into the potential significance of actively maintaining cardiometabolic risk factor control in reducing the risk of non-coronary complications, such as extracranial large artery disease and lower extremity artery disease, in individuals with type 1 diabetes mellitus. The study revealed a notable excess risk for extracranial large artery disease and lower extremity artery disease among individuals with type 1 diabetes mellitus who had poor risk factor control. Interestingly, when all the selected risk factors were within the target range, there was no excess risk of extracranial large artery disease or lower extremity artery disease associated with type 1 diabetes mellitus. This suggests either that people with type 1 diabetes mellitus who maintain good glycemia and other risk factors, do not have elevated risk of extracranial large artery disease or lower extremity artery disease, or that good risk factor control can attenuate excess risk.

The current study results on rates and risk for lower extremity artery disease and diabetic foot disease in persons with type 1 diabetes mellitus are consistent with previous findings. However, this study's point estimates differ somewhat compared with previous results which may be attributed to somewhat different outcome definitions.^31^^,^^32^ extracranial large artery disease is a composite outcome consisting of both carotid and vertebral atherosclerosis. Prior data on extracranial large artery disease is limited, however there is data from the Diabetes Control and Complications trial (DCCT) indicating intensive therapy reduces the progression of intima media thickness, which is in line with present findings.^33^

Study findings suggest that people with type 1 diabetes mellitus, even in presence of selected risk factors that are beyond target levels, have a lower excess risk of incident aortic aneurysm compared with matched controls. Previous studies have also shown a lower risk of aortic aneurysm, presumably due to increased accumulation of advanced glycation end products in tunica media.^34^ The present analyses identified dysglycemia and income level as important factors associated with outcomes, while blood pressure and smoking were less important in the models, which stands in contrast with some previous findings.^34^, ^35^, ^36^ Dysglycemia is regarded as a pro-atherosclerotic condition that leads to aortic stiffening and reduces the likelihood of aneurysm development. Previous studies may not have specifically considered diabetes mellitus or differentiated between type 1 diabetes mellitus and type 2 diabetes mellitus when evaluating the association between risk factors and aortic aneurysm and aortic dissection,^36^, ^37^, ^38^, ^39^, ^40^, ^41^ thus prior data to compare our findings with are limited. Sensitivity analyses of aortic aneurysm revealed no difference between thoracic aortic aneurysm and abdominal aortic aneurysm.

This indicates a potential causal link between dysglycemia and aortic aneurysm risk, as supported by previous observational and experimental studies.^35^^,^^37^^,^^41^ Elevated glycemic load is associated with lower risk for aortic aneurysm but substantially increases risk of non-coronary complications, more so than other cardiometabolic risk factors.^42^ However, it's crucial to interpret this protective effect of type 1 diabetes mellitus on incident aortic aneurysm with caution. The phenotype of vascular damage can vary depending on the part of our vasculature bed that is affected. The apparent protective effect of type 1 diabetes mellitus on incident aortic aneurysm should not be interpreted as a positive outcome, but rather as a manifestation of pathological vascular damage caused by dysglycemia in other vasculature beds, while risk of aortic complications may be reduced. Also, the clear impact of dysglycemia for the development of diabetic nephropathy- and retinopathy, and now diabetic foot disease highlights a potential causal role of glucometabolic pathological processes caused by dysglycemia in several microvascular beds.

### Limitations

A limitation is that cardiovascular risk factor data were not available for controls. Another limitation is the use of imputed baseline values of risk factors in the analyses. However, using baseline values is often preferred from a clinical perspective, as it reflects the initial status of risk factors at the start of the study. The current study design comes with an inherent selection bias. Study participants can contribute observation time for several periods if they do not experience an event. This means that those without events contribute observation time to the following periods. Consequently, there is a sequential accumulation of healthier individuals in each period. As a result, this may result in a cohort progressively comprised of individuals at lower risk. Furthermore, the data in Supplementary Table S4 represents baseline characteristics for each time-period, while Fig. 7 illustrates changes in baseline risk factors based on least-squared means. It's important to note that these data do not depict a sequential accumulation of individuals but instead showcase unique cases registered in each time-period. Additionally, the use of advanced statistical models in our study may introduce model-dependent results. There are very small differences in age and sex distribution between cases with diabetes and controls, as a result of, applying our exclusion criteria. This study did not distinguish between achieving target risk factor levels de novo or through therapeutic interventions. This study adopts a distinctive definition of ICD-codes for different non-coronary complications, which complicates their comparison with existing studies, especially regarding diabetic foot disease. Additionally, the study had an exploratory nature, and the analyses were not corrected for multiple comparisons. Residual confounding, such as lack of data in C-reactive protein, despite multivariable statistical modeling, is also a potential limitation, as with all epidemiological studies. The assessment of change in risk over time is a complex task and various methods can be employed to assess this aspect thoroughly, the methods employed in this manuscript is an estimation of changes in risk over time-periods and the model that incorporates time as a continuous predictor assumes a linear relationship between time and risk, which holds after examining the assumption.

### Conclusions

Over the past two decades, there has been a reduction in the incidence rates of non-coronary complications. Remarkably, when all selected cardiovascular risk factors were within target levels, there was no excess risk of extracranial large artery disease or lower extremity artery disease associated with type 1 diabetes mellitus compared with controls. This suggests that maintaining optimal levels of cardiovascular risk factors, may play an important role in reducing the risk of extracranial large artery disease and lower extremity artery disease in individuals with type 1 diabetes mellitus. Furthermore, there was a graded association between the number of risk factors beyond target levels and the incremental risk of extracranial large artery disease, lower extremity artery disease, and diabetic foot disease among people with type 1 diabetes mellitus. Persons with type 1 diabetes mellitus and dysglycemia appeared to have a reduced effect for aortic aneurysm.

## Contributors

The first three authors (T.A, B.E and Ar.R), as well as the last author (Ai.R) had full access to the data. The study was designed by the last author (Ai.R). The first draft of the manuscript was written by the first author (T.A). The statistical analyses, as well as, preparation of figures and tables were performed by the last author (Ai.R). Ai.R. is the guarantor of this work and, as such, had all access to all the data in the study and takes responsibility for the integrity of the data and the accuracy of the data analysis. All of the authors participated in data analysis and interpretation. All authors made the decision to submit the manuscript for publication. All named authors meet the International Committee of Medical Journal Editors criteria for authorship for this article, take responsibility for the integrity of the work as a whole, and have given their approval for this version to be published.

## Data sharing statement

Because of the sensitive nature of the data collected for this study, access to the datasets is available from the sources stated in the paper on request to the data providers, fulfilling the legal and regulatory requirements, and with approval from the Swedish Ethical Review Authority.

## Declaration of interests

Dr. Bhatt discloses the following relationships - Advisory Board: Angiowave, Bayer, Boehringer Ingelheim, CellProthera, Cereno Scientific, Elsevier Practice Update Cardiology, High Enroll, Janssen, Level Ex, McKinsey, Medscape Cardiology, Merck, MyoKardia, NirvaMed, Novo Nordisk, PhaseBio, PLx Pharma, Stasys; Board of Directors: American Heart Association New York City, Angiowave (stock options), Bristol Myers Squibb (stock), DRS. LINQ (stock options), High Enroll (stock); Consultant: Broadview Ventures, GlaxoSmithKline, Hims, SFJ, Youngene; Data Monitoring Committees: Acesion Pharma, Assistance Publique-Hôpitaux de Paris, Baim Institute for Clinical Research (formerly Harvard Clinical Research Institute, for the PORTICO trial, funded by St. Jude Medical, now Abbott), Boston Scientific (Chair, PEITHO trial), Cleveland Clinic, Contego Medical (Chair, PERFORMANCE 2), Duke Clinical Research Institute, Mayo Clinic, Mount Sinai School of Medicine (for the ENVISAGE trial, funded by Daiichi Sankyo; for the ABILITY-DM trial, funded by Concept Medical; for ALLAY-HF, funded by Alleviant Medical), Novartis, Population Health Research Institute; Rutgers University (for the NIH-funded MINT Trial); Honoraria: American College of Cardiology (Senior Associate Editor, Clinical Trials and News, ACC.org; Chair, ACC Accreditation Oversight Committee), Arnold and Porter law firm (work related to Sanofi/Bristol-Myers Squibb clopidogrel litigation), Baim Institute for Clinical Research (formerly Harvard Clinical Research Institute; RE-DUAL PCI clinical trial steering committee funded by Boehringer Ingelheim; AEGIS-II executive committee funded by CSL Behring), Belvoir Publications (Editor in Chief, Harvard Heart Letter), Canadian Medical and Surgical Knowledge Translation Research Group (clinical trial steering committees), CSL Behring (AHA lecture), Cowen and Company, Duke Clinical Research Institute (clinical trial steering committees, including for the PRONOUNCE trial, funded by Ferring Pharmaceuticals), HMP Global (Editor in Chief, Journal of Invasive Cardiology), Journal of the American College of Cardiology (Guest Editor; Associate Editor), K2P (Co-Chair, interdisciplinary curriculum), Level Ex, Medtelligence/ReachMD (CME steering committees), MJH Life Sciences, Oakstone CME (Course Director, Comprehensive Review of Interventional Cardiology), Piper Sandler, Population Health Research Institute (for the COMPASS operations committee, publications committee, steering committee, and USA national co-leader, funded by Bayer), WebMD (CME steering committees), Wiley (steering committee); Other: Clinical Cardiology (Deputy Editor); Patent: Sotagliflozin (named on a patent for sotagliflozin assigned to Brigham and Women's Hospital who assigned to Lexicon; neither I nor Brigham and Women's Hospital receive any income from this patent); Research Funding: Abbott, Acesion Pharma, Afimmune, Aker Biomarine, Alnylam, Amarin, Amgen, AstraZeneca, Bayer, Beren, Boehringer Ingelheim, Boston Scientific, Bristol-Myers Squibb, Cardax, CellProthera, Cereno Scientific, Chiesi, CinCor, Cleerly, CSL Behring, Eisai, Ethicon, Faraday Pharmaceuticals, Ferring Pharmaceuticals, Forest Laboratories, Fractyl, Garmin, HLS Therapeutics, Idorsia, Ironwood, Ischemix, Janssen, Javelin, Lexicon, Lilly, Medtronic, Merck, Moderna, MyoKardia, NirvaMed, Novartis, Novo Nordisk, Otsuka, Owkin, Pfizer, PhaseBio, PLx Pharma, Recardio, Regeneron, Reid Hoffman Foundation, Roche, Sanofi, Stasys, Synaptic, The Medicines Company, Youngene, 89Bio; Royalties: Elsevier (Editor, Braunwald's Heart Disease); Site Co-Investigator: Abbott, Biotronik, Boston Scientific, CSI, Endotronix, St. Jude Medical (now Abbott), Philips, SpectraWAVE, Svelte, Vascular Solutions; Trustee: American College of Cardiology; Unfunded Research: FlowCo. JN reports no conflicts of interest.

Dr. McGuire reports research support for Clinical Trials Leadership from Boehringer Ingelheim, Merck & Co, Pfizer, AstraZeneca, Novo Nordisk, Esperion, Lilly USA, Lexicon, CSL Behringm New Amsterdam; honoraria for consultancy from Lilly USA, Boehringer Ingelheim, Merck & Co, Novo Nordisk, Applied Therapeutics, Altimmune, CSL Behring, Bayer, GlaxoSmithKline, Intercept. BE reports personal fees from Amgen, AstraZeneca, Boehringer Ingelheim, Eli Lilly, Merck Sharp and Dohme, Mundipharma, NovoNordisk and Sanofi, all outside the submitted work.

The remaining authors have nothing to disclose.
