# Supplementary material for: Non-coronary arterial outcomes in people with type 1 diabetes mellitus: a Swedish retrospective cohort study
Source: Lancet Reg Health Eur. 2024 Feb 15;39:100852. doi: 10.1016/j.lanepe.2024.100852 (PMC11129280; doi:10.1016/j.lanepe.2024.100852)
Supplement: Supplementary Tables S1–S6 and Supplementary Figures S1–S3 [file mmc1.pdf]

SUPPLEMENTAL MATERIAL

Non-coronary arterial outcomes in people with type 1 diabetes mellitus: a Swedish retrospective cohort study

Table S1. ICD-10 Codes Used to Define Baseline Conditions and Outcomes		
**Outcomes**		**ICD-10**
Lower extremity artery disease		I702, I702A, I702C, I702D, I702X, I702E, I739B, 1700
Diabetic foot disease		E105, E105A, E105B, E105W, E105X, E1051, E106D
Extracranial large artery disease		I630, I631, I632, I635, I652, I650, I659, I653, I658
Aortic aneurysm		I714, I719, I713, I712, I711, I716, I715, I718
Aortic dissection		I710B, I710, I710X, I710W, I710A
**Comorbidities**		
Hypertension		I109, I11, I120, I129, I132, I139, I130, I131, I159, I150, I152, I158
Dementia		F00, F01, F02, F039 N179, N178, N170, N172, N189, N181, N182, N183, N184, N185, N199, Z940, DR016, N083, E112C
End-stage renal disease		E112C, E112W, E112X, E102, E102X, E132, E242, E122, E112A, E112B, I120, Z992, DR013, DR023, DR056, Z941, Z492
Cancer		C00–C97
COPD		J44
Atrial fibrillation		I489, I480, I481, I482, I483, I483
Heart failure		I50
Ischemic heart disease		I20-I25
Acute myocardial infarction		I214, I219, I210, I211, I213, I212
Cerebrovascular disease		I61-I64

* Includes ICD-codes as main diagnosis and up to 6 contributory causes for outcomes and comorbidities. Abbreviations: COPD – Chronic pulmonary obstructive disease

Table S2. Variables Used in the Imputation Algorithm.

Age, sex, age when diabetes debuted, insulin treatment, systolic blood pressure, diastolic blood pressure, body weight, body length, glycated hemoglobin (HbA1c), total cholesterol, triglycerides, high-density lipoprotein cholesterol (HDL-C), low-density lipoprotein cholesterol (LDL-C), albuminuria, s-creatinine, retinopathy, smoking status, physical activity, county, body mass index, marital status, education, ethnicity, income, estimated glomerular filtration rate (eGFR), comorbidities at baseline (acute myocardial infarction, coronary heart disease, heart failure, hypertension, chronic obstructive pulmonary disease, dementia, end-stage renal disease, cancer, stroke, atrial fibrillation), medication at baseline (treatment with anti-hypertensive medication, statins, antithrombotic and anti-coagulant medication), lower extremity artery disease, extracranial large artery disease, aortic aneurysm, diabetic foot syndrome

Table S3. Baseline characteristics for all patients with type 1 diabetes and all matched controls.

	Diabetes	Controls
**n**	34 263	164 063
**Sex = female %)**	14 826 (43.3)	71 922 (43.8)
**Age (mean (SD))**	33.35 (16.23)	32.05 (15.06)
**Age-category (%)**		
**<45**	26 215 (76.5)	130 838 (79.7)
**45-54**	2 649 (7.7)	12 025 (7.3)
**55-64**	3 999 (11.7)	17 284 (10.5)
**65-74**	899 (2.6)	2 677 (1.6)
**>75**	501 (1.5)	1 239 (0.9)
**Education n (%)**		
**Post-secondary education ≥ 12 years**	6 227 (18.2)	33 126 (20.2)
**Pre-secondary education ≤ 9 years**	14 818 (43.2)	68 207 (41.6)
**Secondary education >9 to 12 years**	13 218 (38.6)	62 730 (38.2)
**Civil status: married n (%)**	6 672 (19.5)	30 104 18.3)
**Ethnicity = Scandinavian, n (%)**	31 247 (91.2)	147 658 (90.0)
**Income family interquartile range (IQR)) n (%)**		
**IQR 1**	8 199 (23.9)	34 083 (20.8)
**IQR 2**	7 008 (20.5)	33 616 (20.5)
**IQR 3**	8 883 (25.9)	43 739 (26.7)
**IQR4**	10 173 (29.7)	52 625 (32.1)
**Income (interquartile range (IQR)) n (%)**		
**IQR 1**	18 233 (53.2)	83 449 (50.9)
**IQR 2**	6 435 (18.8)	28 532 (17.4)
**IQR 3**	6 480 (18.9)	33 715 (20.6)
**IQR4**	3 115 (9.1)	18 367 (11.2)
**Hypertension n (%)**	2061 (6.0)	1 592 (1.0)
**Chronic obstructive pulmonary disease n (%)**	98 (0.3)	257 (0.2)
**Ischemic heart disease n (%)**	754 (2.2)	802 (0.5)
**Heart failure n (%)**	321 (0.9)	287 (0.2)
**Cerebrovascular disease n (%)**	215 (0.6)	375 (0.2)
**Dementia n (%)**	39 (0.1)	95 (0.1)
**End-stage renal disease n (%)**	1 169 (3.4)	246 (0.1)
**Cancer n (%)**	576 (1.7)	1 921 (1.2)
**Antihypertensive medication n (%)**	8 859 (25.9)	17 151 (10.5)
**Statins n (%)**	9 512 (27.8)	6 253 (3.8)
**Antiocoagulant medication n (%)**	801 (2.3)	3 269 (2.0)
**Antithrombotic medication n (%)**	2 914 (8.5)	3 879 (2.4)
**Age at onset of diabetes (mean (SD))**	18.67 (12.21)	

**Duration of diabetes (mean (SD))**	12.60 (12.45)	
**Glycated hemoglobin levels (mean (SD)) (mmol/mol)**	65.27 (18.58)	
**Current smoking n (%)**	5 163 (15.1)	
**Albuminuria n (%)**		
**No albuminuria**	30 372 (88.6)	
**Normal albuminuria**	97 (0.3)	
**Microalbuminuria**	2 585 (7.5)	
**Macroalbuminuria**	1 209 (3.5)	
**Estimated glomerular filtration rate (eGFR) (mean (SD))**	117.07 (57.16)	
**Retinopathy n (%)**	10 435 (30.5)	
**Systolic blood pressure (mean (SD)) (mmHg)**	122.91 (15.27)	
**Diastolic blood pressure (mean (SD)) (mmHg)**	72.95 (9.13)	
**Total cholesterol (mean (SD)) (mg/dL)**	183.13 (42.05)	
**High-density lipoprotein cholesterol (mean (SD)) (mg/dL)**	62.02 (19.75)	
**Triglycerides (mean (SD)) (mg/dL)**	118.32 (112.40)	
**Low-density lipoprotein cholesterol (mean (SD)) (mg/dL)**	100.29 (34.86)	
**Physical activity n (%)**		
**1=Never**	3 835 (11.2)	
**2=<1 time/week**	4 730 (13.8)	
**3= 1-2 times/week**	7 260 (21.2)	
**4= 3-5 times/week**	9 374 (27.4)	
**5=Daily**	9 064 (26.5)	
**S-creatinine (mean (SD)) (µmol/L)**	70.99 (37.98)	
**Body mass index (mean (SD)) (kg/m2)**	25.04 (4.18)	

Table S4. Baseline characteristics for patients with type 1 diabetes according to time-period.									
Time-period	**2001-2002**	**2003-2004**	**2005-2006**	**2007-2008**	**2009-2010**	**2011-2012**	**2013-2014**	**2015-2016**	**2017-2019**
n	4830	4318	4017	3648	3479	4157	2780	2870	4164
Sex = female %)	2089 (43.3)	1897 (43.9)	1773 (44.1)	1633 (44.8)	1457 (41.9)	1710 (41.1)	1217 (43.8)	1250 (43.6)	1800 (43.2)
Age (mean (SD))	38.18 (14.13)	36.24 (14.81)	35.75 (15.42)	32.95 (16.73)	31.15 (17.46)	29.93 (15.20)	25.87 (12.17)	26.95 (13.71)	37.41 (19.53)
**Education n (%)**									
Post-secondary education ≥ 12 years	1210 (25.1)	990 (22.9)	913 (22.7)	658 (18.0)	494 (14.2)	796 (19.1)	381 (13.7)	403 (14.0)	382 (9.2)
Pre-secondary education ≤ 9 years	1070 (22.2)	1072 (24.8)	1087 (27.1)	1376 (37.7)	1689 (48.5)	1990 (47.9)	1655 (59.5)	1723 (60.0)	3156 (75.8)
Secondary education >9 to 12 years	2550 (52.8)	2256 (52.2)	2017 (50.2)	1614 (44.2)	1296 (37.3)	1371 (33.0)	744 (26.8)	744 (25.9)	626 (15.0)
Civil status: married n (%)	1650 (34.2)	1240 (28.7)	1031 (25.7)	695 (19.1)	512 (14.7)	686 (16.5)	287 (10.3)	294 (10.2)	277 (6.7)
Ethnicity = Scandinavian, n (%)	4656 (96.4)	4126 (95.6)	3756 (93.5)	3346 (91.7)	3162 (90.9)	3822 (91.9)	2455 (88.3)	2456 (85.6)	3468 (83.3)
**Income family interquartile range (IQR)) n (%)**									
Quartile 1	1686 (34.9)	1400 (32.4)	1259 (31.3)	994 (27.2)	803 (23.1)	698 (16.8)	426 (15.3)	480 (16.7)	453 (10.9)
Quartile 2	1384 (28.7)	1065 (24.7)	972 (24.2)	800 (21.9)	732 (21.0)	759 (18.3)	477 (17.2)	419 (14.6)	400 (9.6)
Quartile 3	1234 (25.5)	1187 (27.5)	1114 (27.7)	872 (23.9)	708 (20.4)	813 (19.6)	537 (19.3)	542 (18.9)	1876 (45.1)
Quartile 4	526 (10.9)	666 (15.4)	672 (16.7)	982 (26.9)	1236 (35.5)	1887 (45.4)	1340 (48.2)	1429 (49.8)	1435 (34.5)
**Income (interquartile range (IQR)) n (%)**									
Quartile 1	1979 (41.0)	1870 (43.3)	1894 (47.1)	2027 (55.6)	2219 (63.8)	2474 (59.5)	1910 (68.7)	1918 (66.8)	1942 (46.6)
Quartile 2	1690 (35.0)	1331 (30.8)	1077 (26.8)	727 (19.9)	452 (13.0)	408 (9.8)	264 (9.5)	273 (9.5)	213 (5.1)
Quartile 3	850 (17.6)	846 (19.6)	748 (18.6)	592 (16.2)	486 (14.0)	573 (13.8)	322 (11.6)	377 (13.1)	1686 (40.5)
Quartile 4	311 (6.4)	271 (6.3)	298 (7.4)	302 (8.3)	322 (9.3)	702 (16.9)	284 (10.2)	302 (10.5)	323 (7.8)
Hypertension n (%)	221 (4.6)	266 (6.2)	301 (7.5)	297 (8.1)	314 (9.0)	303 (7.3)	106 (3.8)	128 (4.5)	125 (3.0)
Ischemic heart disease n (%)	140 (2.9)	134 (3.1)	126 (3.1)	130 (3.6)	108 (3.1)	66 (1.6)	16 (0.6)	18 (0.6)	16 (0.4)
Heart failure n (%)	35 (0.7)	46 (1.1)	42 (1.0)	60 (1.6)	76 (2.2)	31 (0.7)	9 (0.3)	8 (0.3)	14 (0.3)
Cerebrovascular disease n (%)	28 (0.6)	33 (0.8)	27 (0.7)	32 (0.9)	39 (1.1)	27 (0.6)	12 (0.4)	9 (0.3)	8 (0.2)
Chronic obstructive pulmonary disease n (%)	3 (0.1)	6 (0.1)	15 (0.4)	25 (0.7)	25 (0.7)	12 (0.3)	6 (0.2)	3 (0.1)	3 (0.1)
Dementia n (%)	1 (0.0)	1 (0.0)	5 (0.1)	10 (0.3)	12 (0.3)	2 (0.0)	3 (0.1)	4 (0.1)	1 (0.0)
End-stage renal disease n (%)	194 (4.0)	202 (4.7)	223 (5.6)	156 (4.3)	130 (3.7)	117 (2.8)	50 (1.8)	39 (1.4)	58 (1.4)
Cancer n (%)	57 (1.2)	64 (1.5)	76 (1.9)	79 (2.2)	79 (2.3)	85 (2.0)	33 (1.2)	51 (1.8)	52 (1.2)
Antihypertensive medication n (%)	2188 (45.3)	1743 (40.4)	1462 (36.4)	990 (27.1)	720 (20.7)	831 (20.0)	330 (11.9)	286 (10.0)	309 (7.4)

Statins n (%)	2418 (50.1)	1955 (45.3)	1547 (38.5)	1086 (29.8)	752 (21.6)	854 (20.5)	341 (12.3)	287 (10.0)	272 (6.5)
Antiocoagulant medication n (%)	170 (3.5)	145 (3.4)	122 (3.0)	96 (2.6)	56 (1.6)	89 (2.1)	27 (1.0)	43 (1.5)	53 (1.3)
Antithrombotic medication n (%)	794 (16.4)	573 (13.3)	492 (12.2)	352 (9.6)	224 (6.4)	234 (5.6)	90 (3.2)	81 (2.8)	74 (1.8)
Age at onset of diabetes (mean (SD))	19.59 (11.20)	19.43 (11.43)	19.69 (11.72)	19.86 (13.36)	19.07 (14.39)	17.69 (12.29)	17.06 (11.60)	17.81 (12.36)	17.14 (11.34)
Duration of diabetes (mean (SD))	18.44 (13.76)	16.57 (13.79)	15.67 (13.89)	12.63 (12.40)	11.34 (11.77)	11.59 (11.83)	7.99 (8.61)	7.71 (8.39)	7.25 (7.69)
Glycated hemoglobin levels (mean (SD)) (mmol/mol)	64.44 (15.90)	64.89 (16.73)	64.06 (17.05)	64.79 (18.18)	66.63 (19.28)	67.24 (19.21)	65.56 (20.23)	65.57 (20.48)	64.71 (20.93)
Current smoking n (%)	636 (13.2)	643 (14.9)	612 (15.2)	516 (14.1)	543 (15.6)	646 (15.5)	456 (16.4)	466 (16.2)	645 (15.5)
**Albuminuria n (%)**									
No albuminuria	4027 (83.4)	3703 (85.8)	3396 (84.5)	3206 (87.9)	3082 (88.6)	3825 (92.0)	2595 (93.3)	2655 (92.5)	3883 (93.3)
Normal albuminuria	11 (0.2)	3 (0.1)	1 (0.0)	24 (0.7)	14 (0.4)	15 (0.4)	7 (0.3)	11 (0.4)	11 (0.3)
Microalbuminuria	490 (10.1)	413 (9.6)	431 (10.7)	292 (8.0)	250 (7.2)	232 (5.6)	126 (4.5)	148 (5.2)	203 (4.9)
Macroalbuminuria	302 (6.3)	199 (4.6)	189 (4.7)	126 (3.5)	133 (3.8)	85 (2.0)	52 (1.9)	56 (2.0)	67 (1.6)
Estimated glomerular filtration rate (eGFR) (mean (SD))	106.04 (52.31)	96.84 (38.86)	105.07 (44.31)	113.54 (49.70)	119.83 (52.66)	124.34 (62.49)	132.73 (65.66)	131.34 (66.12)	135.62 (67.51)
Retinopathy n (%)	1991 (41.2)	1630 (37.7)	1585 (39.5)	1161 (31.8)	1019 (29.3)	1154 (27.8)	530 (19.1)	580 (20.2)	785 (18.9)
Systolic blood pressure (mean (SD)) (mmHg)	127.52 (16.82)	126.06 (16.31)	125.03 (16.28)	122.59 (14.79)	121.88 (14.70)	120.77 (13.57)	119.77 (13.53)	120.09 (13.60)	119.53 (13.63)
Diastolic blood pressure (mean (SD)) (mmHg)	74.25 (8.77)	73.94 (9.10)	72.78 (9.41)	72.52 (9.23)	72.14 (9.12)	72.39 (9.00)	72.30 (9.23)	72.66 (9.13)	72.81 (9.09)
Total cholesterol (mean (SD)) (mg/dL)	4.89 (1.12)	4.83 (1.05)	4.76 (1.05)	4.75 (1.09)	4.77 (1.12)	4.71 (1.05)	4.64 (1.11)	4.63 (1.10)	4.55 (1.07)
High-density lipoprotein cholesterol (mean (SD)) (mg/dL)	1.78 (0.56)	1.66 (0.52)	1.62 (0.51)	1.59 (0.50)	1.55 (0.51)	1.55 (0.48)	1.54 (0.48)	1.55 (0.47)	1.52 (0.46)
Triglycerides (mean (SD)) (mg/dL)	1.39 (1.46)	1.31 (1.11)	1.24 (1.10)	1.31 (1.15)	1.37 (1.16)	1.33 (1.09)	1.31 (1.27)	1.34 (1.28)	1.37 (1.58)
**Physical activity n (%)**									
1=Never	665 (13.8)	636 (14.7)	466 (11.6)	362 (9.9)	381 (11.0)	368 (8.9)	261 (9.4)	276 (9.6)	420 (10.1)
2=<1 time/week	698 (14.5)	551 (12.8)	529 (13.2)	436 (12.0)	489 (14.1)	710 (17.1)	392 (14.1)	413 (14.4)	512 (12.3)
3= 1-2 times/week	975 (20.2)	847 (19.6)	949 (23.6)	810 (22.2)	749 (21.5)	911 (21.9)	568 (20.4)	642 (22.4)	809 (19.4)
4= 3-5 times/week	1063 (22.0)	965 (22.3)	1039 (25.9)	1048 (28.7)	995 (28.6)	1176 (28.3)	919 (33.1)	850 (29.6)	1319 (31.7)
5=Daily	1429 (29.6)	1319 (30.5)	1034 (25.7)	992 (27.2)	865 (24.9)	992 (23.9)	640 (23.0)	689 (24.0)	1104 (26.5)
Insulin method = 2 (%)	482 (10.0)	445 (10.4)	458 (11.5)	571 (15.8)	578 (16.7)	800 (19.4)	564 (20.4)	645 (22.6)	1124 (27.2)
Low-density lipoprotein cholesterol (mean (SD)) (mg/dL)	2.57 (0.97)	2.62 (0.88)	2.63 (0.89)	2.62 (0.91)	2.66 (0.92)	2.60 (0.88)	2.57 (0.87)	2.56 (0.89)	2.51 (0.87)

S-creatinine (mean (SD)) (µmol/L)	77.58 (53.68)	80.01 (43.53)	74.36 (35.14)	71.22 (34.46)	69.46 (32.48)	67.08 (26.92)	64.67 (33.28)	65.07 (34.97)	63.96 (29.71)
Body mass index (mean (SD)) (kg/m2)	25.13 (3.71)	25.11 (3.93)	25.11 (4.19)	25.12 (4.22)	24.85 (4.16)	24.98 (4.28)	24.89 (4.42)	24.98 (4.37)	25.13 (4.53)

Table S5. Crude- and standardized incidence rates for large- and small-vessel artery disease among both patients with type 1 diabetes and matched controls							
**Event / Period**	**Category**	**Events**	**Person-years**	**Crude rate**	**Adj rate**	**Adj - LCI**	**Adj - UCI**
**ELAD / [2001,2002]**	Diabetes	4	5 107.4	78.3	87.1	20.9	968.4
**ELAD / [2003,2004]**	Diabetes	18	13 871.9	129.8	296.5	149	600.9
**ELAD / [2005,2006]**	Diabetes	19	21 995.5	86.4	183.8	92	358.2
**ELAD / [2007,2008]**	Diabetes	18	29 258.1	61.5	139.4	71.6	259.3
**ELAD / [2009,2010]**	Diabetes	25	35 777.4	69.9	173.3	104.9	276.3
**ELAD / [2011,2012]**	Diabetes	24	42 945.3	55.9	105.7	62.7	177.6
**ELAD / [2013,2014]**	Diabetes	19	48 708.8	39	89	46.7	163
**ELAD / [2015,2016]**	Diabetes	24	53 574.8	44.8	83.6	49.4	143.8
**ELAD / [2017,2019]**	Diabetes	40	89 769	44.6	84.3	56.7	125.5
**ELAD / [2001,2002]**	Controls	3	24 218	12.4	68.5	14.1	454.4
**ELAD / [2003,2004]**	Controls	18	66 198.4	27.2	164.2	79	312.4
**ELAD / [2005,2006]**	Controls	16	105 319.9	15.2	45.6	19.4	108.3
**ELAD / [2007,2008]**	Controls	28	140 392.6	19.9	74.2	41	130.9
**ELAD / [2009,2010]**	Controls	33	172 531.3	19.1	61.8	37.6	101
**ELAD / [2011,2012]**	Controls	28	209 104.8	13.4	55.5	32.3	90.8
**ELAD / [2013,2014]**	Controls	42	239 915.4	17.5	55.8	36.9	82.5
**ELAD / [2015,2016]**	Controls	32	266 158.5	12	29.9	19.2	45.2
**ELAD / [2017,2019]**	Controls	51	448 854.6	11.4	28.7	20.9	38.8
**AA / [2001,2002]**	Diabetes	0	5 109.6	0	0	N/A	903.3
**AA / [2003,2004]**	Diabetes	0	13 895.6	0	0	N/A	229.6
**AA / [2005,2006]**	Diabetes	1	22 046.8	4.5	6.3	0.2	124.1
**AA / [2007,2008]**	Diabetes	2	29 339.6	6.8	11.7	1.1	85.6
**AA / [2009,2010]**	Diabetes	6	35 873.3	16.7	25.1	5.7	83.5
**AA / [2011,2012]**	Diabetes	13	43 063.2	30.2	69.2	31.6	139.1
**AA / [2013,2014]**	Diabetes	3	48 841.4	6.1	21.8	2.9	80

**AA / [2015,2016]**	Diabetes	5	53 713.7	9.3	12.6	2.1	55.7
**AA / [2017,2019]**	Diabetes	6	89 997.3	6.7	9.2	3	31.6
**AA / [2001,2002]**	Controls	3	24 220.9	12.4	100.4	9.6	528.1
**AA / [2003,2004]**	Controls	7	66 206.5	10.6	65.2	17	182.5
**AA / [2005,2006]**	Controls	15	105 352.2	14.2	41.5	16.1	104.3
**AA / [2007,2008]**	Controls	21	140 430.2	15	61.3	30.1	117.3
**AA / [2009,2010]**	Controls	32	172 567.9	18.5	55.2	31.7	94.1
**AA / [2011,2012]**	Controls	38	209 117.9	18.2	58.1	35.1	92.7
**AA / [2013,2014]**	Controls	58	239 916.6	24.2	70.5	49.6	98.8
**AA / [2015,2016]**	Controls	67	266 107.8	25.2	68.1	50.8	90.2
**AA / [2017,2019]**	Controls	97	448 656.4	21.6	40.0	31.7	50.2
**AD / [2001,2002]**	Diabetes	0	5 109.6	0	0	N/A	9 033.1
**AD / [2003,2004]**	Diabetes	0	13 895.6	0	0	N/A	2 296
**AD / [2005,2006]**	Diabetes	0	22 047.7	0	0	N/A	1 186.6
**AD / [2007,2008]**	Diabetes	0	29 343.1	0	0	N/A	726.7
**AD / [2009,2010]**	Diabetes	0	35 885.7	0	0	N/A	483.3
**AD / [2011,2012]**	Diabetes	0	43 096	0	0	N/A	434.1
**AD / [2013,2014]**	Diabetes	3	48 875.6	61.4	68.9	11.2	504
**AD / [2015,2016]**	Diabetes	2	53 744.8	37.2	39.7	3.7	429.2
**AD / [2017,2019]**	Diabetes	0	90 054.3	0	0	N/A	211.2
**AD / [2001,2002]**	Controls	3	24 221.3	123.9	311.9	36.5	4 145.1
**AD / [2003,2004]**	Controls	3	66 210.4	45.3	62.2	12.6	865.6
**AD / [2005,2006]**	Controls	6	105 365.3	56.9	101.4	30.1	596.8
**AD / [2007,2008]**	Controls	1	140 466.4	7.1	12.6	0.3	347.1
**AD / [2009,2010]**	Controls	9	172 636	52.1	90.1	38.7	313.2
**AD / [2011,2012]**	Controls	8	209 248.5	38.2	91.9	28.7	277.9
**AD / [2013,2014]**	Controls	4	240 103.1	16.7	32.6	8.9	142.8
**AD / [2015,2016]**	Controls	11	266 393.1	41.3	69.7	32.3	155

**AD / [2017,2019]**	Controls	12	449 228.9	26.7	63.8	30.8	121
**LEAD / [2001,2002]**	Diabetes	8	5106	156.7	456.5	111.6	1 520.4
**LEAD / [2003,2004]**	Diabetes	24	13 859.9	173.2	408.9	223.2	751.5
**LEAD / [2005,2006]**	Diabetes	40	21 969.7	182.1	377.6	251.3	576.8
**LEAD / [2007,2008]**	Diabetes	83	29 145.4	284.8	723.8	546.2	951
**LEAD / [2009,2010]**	Diabetes	83	35 573.7	233.3	594.1	457.6	764.7
**LEAD / [2011,2012]**	Diabetes	97	42 685.7	227.2	546.4	429.3	692.3
**LEAD / [2013,2014]**	Diabetes	85	48 373.2	175.7	416.6	317	545.7
**LEAD / [2015,2016]**	Diabetes	73	53 198.1	137.2	353	263.7	469.4
**LEAD / [2017,2019]**	Diabetes	130	89 126.7	145.9	311.1	254.1	381.3
**LEAD / [2001,2002]**	Controls	6	24 219.4	24.8	66.1	17.7	446.7
**LEAD / [2003,2004]**	Controls	11	66 200.9	16.6	39	16.8	122.2
**LEAD / [2005,2006]**	Controls	18	105 325.3	17.1	50.7	23.3	113.6
**LEAD / [2007,2008]**	Controls	28	140 400.2	19.9	90.2	51.1	153.4
**LEAD / [2009,2010]**	Controls	38	172 538.4	22	77.5	48.4	121.8
**LEAD / [2011,2012]**	Controls	40	209 103.1	19.1	66.9	42.7	102.3
**LEAD / [2013,2014]**	Controls	34	239 908.4	14.2	49.8	31.3	76.4
**LEAD / [2015,2016]**	Controls	52	266 153.2	19.5	62.6	45	85.3
**LEAD / [2017,2019]**	Controls	91	448 748.2	20.3	53.7	42.7	67
**DFD/ [2001,2002]**	Diabetes	19	5 099.4	372.6	814.7	367.9	1 894.3
**DFD/ [2003,2004]**	Diabetes	30	13 818.9	217.1	411.9	229.8	750.5
**DFD/ [2005,2006]**	Diabetes	44	21 894.9	201	340.3	216.2	540.4
**DFD / [2007,2008]**	Diabetes	75	29 063.3	258.1	530.4	393.6	713.1
**DFD / [2009,2010]**	Diabetes	55	35 492.6	155	310.1	222	430.2
**DFD/ [2011,2012]**	Diabetes	50	42 637.7	117.3	228.2	161.5	322.6
**DFD / [2013,2014]**	Diabetes	44	48 382.7	90.9	209.6	141.8	307.4
**DFD/ [2015,2016]**	Diabetes	36	53 262.1	67.6	150.3	96.5	231.3
**DFD/ [2017,2019]**	Diabetes	32	89 364	35.8	77.6	49.5	120.2

Abbreviations: ELAD= Extracranial large artery disease. AA=Aortic Aneurysm, LEAD=Lower extremity artery disease, AD= Aortic Dissection, DFD= Diabetic foot disease, UCI=Upper Confidence Interval, LCI= Lower Confidence Interval

Table S6. Excess risk of ELAD, AA, AD and LEAD with adjustment for age, sex, comorbidities, medication and socioeconomic variables in patients with type 1 diabetes												
Characteristic	ELAD			AA			LEAD			AD		
HR[1]	HR[1]	95% CI[1]	p-value	HR[1]	95% CI[1]	p-value	HR[1]	95% CI[1]	p-value	HR[1]	95% CI[1]	p-value
Category												
Controls	—	—		—	—		—	—		—	—	
Type 1 diabetes	1.88	1.53, 2.31	<0.001	0.29	0.20, 0.41	<0.001	6.28	5.42, 7.27	<0.001	0.36	0.14, 0.94	0.037
Age	1.08	1.07, 1.09	<0.001	1.07	1.06, 1.08	<0.001	1.08	1.08, 1.09	<0.001	1.06	1.04, 1.08	<0.001
Sex	0.73	0.59, 0.89	<0.001	0.34	0.26, 0.44	<0.001	0.78	0.61, 0.81	<0.001	0.56	0.32, 0.97	<0.001
Ethnicity												
All other countries	—	—		—	—		—	—		—	—	
Scandinavia	2.03	1.25, 3.31	0.004	1.36	0.88, 2.09	0.2	1.04	0.81, 1.34	0.7	0.52	0.26, 1.08	0.079
Civil												
All other marital statuses	—	—		—	—		—	—		—	—	
Married	0.91	0.75, 1.11	0.3	0.97	0.78, 1.21	0.8	0.93	0.81, 1.07	0.3	0.80	0.46, 1.38	0.4
Education												
Post-secondary education ≥ 12 years	—	—		—	—		—	—		—	—	
Pre-secondary education ≤ 9 years	1.12	0.84, 1.50	0.4	1.20	0.87, 1.63	0.3	1.27	1.04, 1.55	0.018	0.98	0.47, 2.03	>0.9
Secondary education >9 to 12 years	1.32	1.02, 1.72	0.035	1.31	0.99, 1.74	0.062	1.35	1.12, 1.63	0.002	1.06	0.54, 2.06	0.9
Income IQR												
IQR 1	—	—		—	—		—	—		—	—	

**IQR 2**	0.98	0.77, 1.24	0.8	0.88	0.67, 1.15	0.3	1.05	0.89, 1.22	0.6	1.04	0.53, 2.03	>0.9
**IQR 3**	0.56	0.42, 0.75	<0.001	0.63	0.47, 0.85	0.002	0.65	0.54, 0.79	<0.001	0.92	0.46, 1.81	0.8
**IQR 4**	0.85	0.61, 1.18	0.3	0.84	0.60, 1.17	0.3	0.65	0.50, 0.84	<0.001	0.76	0.30, 1.88	0.5
**Comorbidities**												
Hypertension	1.32	0.98, 1.77	0.068	1.46	1.01, 2.12	0.045	1.56	1.30, 1.87	<0.001	0.46	0.10, 2.01	0.3
Ischemic heart disease	0.90	0.63, 1.29	0.6	1.33	0.88, 2.01	0.2	1.33	1.07, 1.64	0.009	0.79	0.17, 3.58	0.8
Heart failure	2.22	1.26, 3.91	0.006	1.30	0.56, 3.01	0.5	2.22	1.63, 3.02	<0.001	2.24	0.28, 18.1	0.5
Stroke	1.66	1.01, 2.72	0.046	1.72	0.90, 3.30	0.10	1.18	0.83, 1.70	0.4	1.67	0.22, 12.8	0.6
**Medication**												
Antihypertensives	0.52	0.41, 0.67	<0.001	1.62	1.23, 2.13	<0.001	0.56	0.47, 0.67	<0.001	2.09	1.10, 3.95	0.024
Statins	1.87	1.43, 2.44	<0.001	1.36	1.03, 1.79	0.032	1.09	0.91, 1.30	0.4	0.61	0.29, 1.29	0.2
Anticoagulants	1.59	1.18, 2.15	0.002	3.18	2.46, 4.12	<0.001	2.00	1.63, 2.45	<0.001	3.14	1.59, 6.18	<0.001
Antithrombotics	6.78	5.25, 8.76	<0.001	2.49	1.90, 3.27	<0.001	4.60	3.87, 5.46	<0.001	2.81	1.37, 5.79	0.005

[1] HR = Hazard Ratio, CI = Confidence Interval, ELAD= Extracranial large artery disease, AA= Aortic aneurysm, LEAD= Lower extremity artery disease, AD= Aortic dissection
***Based on a Cox regression with adjustment for age, comorbidities, mediciation and socioeconomic variables, and stratified for sex**

Supplementary Figure S1. Flowchart for study participants

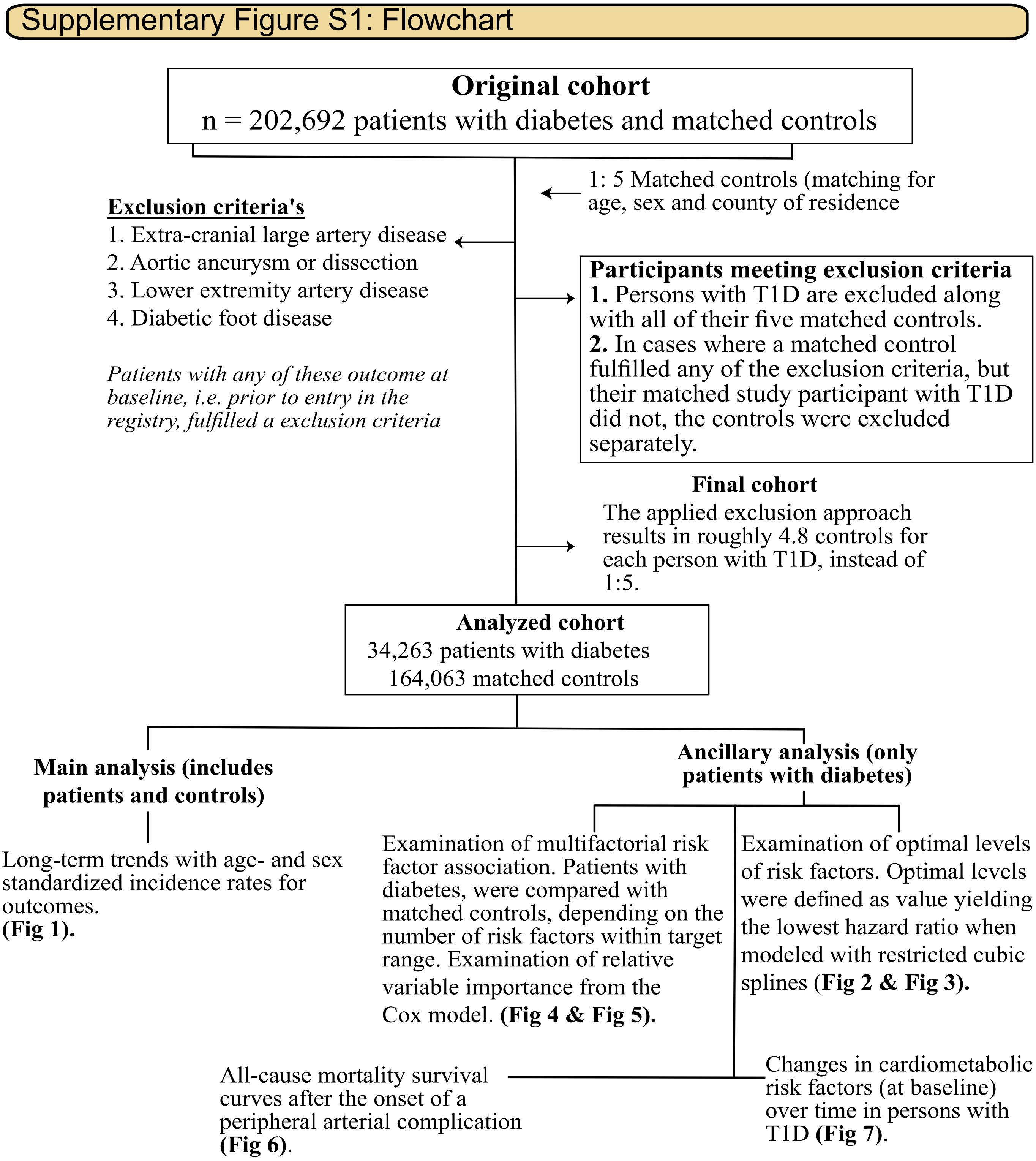

Legend: Study participants may fulfill more than one exclusion criteria

Supplementary Figure S2. Changes in risk over time for non-coronary complications using Cox regression, while adjusting for age, sex and time period.

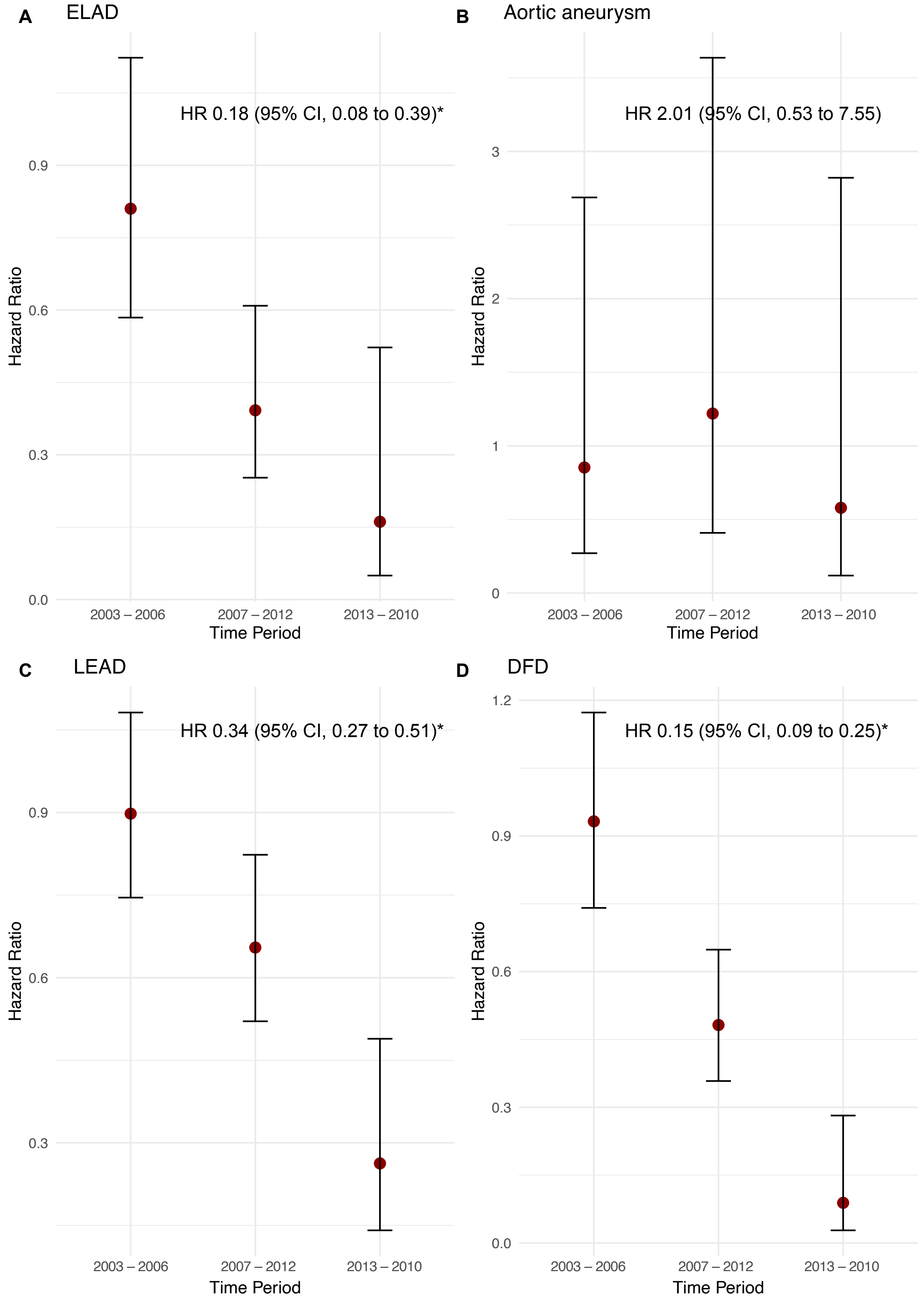

Figure legend: Each figure panel displays results from the Cox regression model, with adjustment for age, sex and time period as a categorical variable with 4 levels. In the upper right corner displays the hazard ratio from another Cox regression, using time period as a continuous variable and all 9 time periods, the coefficients are later on raised to the power of 8 to yield relative risk reduction over roughly an 18-years period.

Supplementary Figure S3. Analyses of abdominal – and thoracic aortic aneurysm in people with people with type 1 diabetes

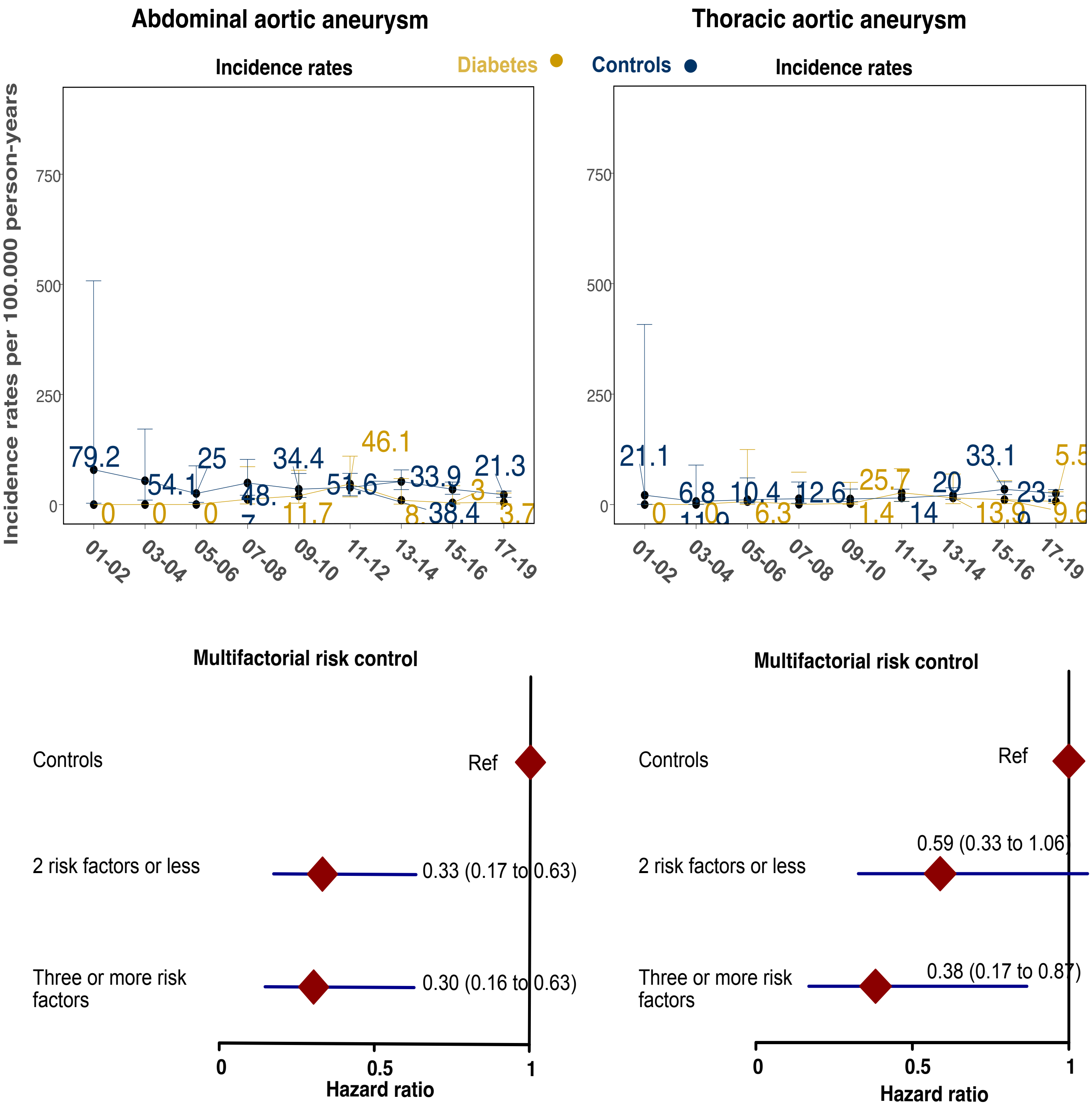

Excess risk for type 1 diabetes in entire cohort

AAA: HR 0.31 (95% CI, 0.19 to 0.52)

TAA: HR 0.49 (95% CI, 0.30 to 0.81)

Figure legend: Incidence rates for AAA and TAA are presented as well as Cox regression with multifactorial risk factor control. Cox regression models for AAA was adjusted for age, sex and socioeconomic variables, whereas regression models for TAA was adjusted for age, sex, socioeconomic variables and comorbidities.
